# Sicurpest: A Prototype of a User-Friendly Tool for Preventive Risk Assessment of Pesticide Use in Agriculture

**DOI:** 10.3390/toxics13020089

**Published:** 2025-01-24

**Authors:** Federico Maria Rubino, Daniele Puri, Mario Fargnoli, Mara Lombardi, Stefan Mandić-Rajčević, Claudio Colosio

**Affiliations:** 1 Department of Health Sciences, University of Milano, Via A Di Rudinì 8, 20124 Milano, Italy; federico.rubino@unimi.it; 2 Department of Technological Innovations and Safety of Plants, Products and Anthropic Settlements (DIT), Italian Workers’ Compensation Authority (INAIL), Via Fontana Candida 1, Monte Porzio Catone, 00078 Roma, Italy; d.puri@inail.it; 3 Department of Mechanical and Aerospace Engineering, Sapienza University of Rome, Via Eudossiana 18, 00184 Rome, Italy; mario.fargnoli@uniroma1.it; 4 Department of Chemical Engineering Materials Environment (DICMA), Sapienza University of Rome, Via Eudossiana 18, 00184 Rome, Italy; mara.lombardi@uniroma1.it; 5 Institute of Social Medicine, Faculty of Medicine, University of Belgrade, 11000 Belgrade, Serbia; stefan.mandic-rajcevic@med.bg.ac.rs; 6 School of Public Health and Health Management, Faculty of Medicine, University of Belgrade, 11000 Belgrade, Serbia; 7 Post Graduate School in Occupational Health, University of Milano, 20124 Milano, Italy

**Keywords:** agriculture, occupational health and safety (OHS), risk management, chemical risks, pesticide exposure, risk assessment, Sicurpest software

## Abstract

In recent years, there has been a significant increase in attention towards the use of pesticides, as evidenced by the introduction of regulations aimed at ensuring their safe and environmentally responsible application. Although this stricter legislative framework offers potential benefits, the challenges faced by farmers, particularly those in small-scale operations, in complying with occupational health and safety (OHS) requirements are considerable. To address this issue, a research project was promoted by the Italian Workers’ Compensation Authority (INAIL) aimed at developing a user-friendly software tool to support farmers in planning pesticide applications in safe conditions. This study summarizes the results of the research, which consisted of three main phases: the definition of the main parameters that characterize the farmers’ exposure based on the analysis of the literature; the development of a risk assessment model that integrates these determinants with data provided by producers for each authorized pesticide; and the development of software tool (called SICURPEST) for a preliminary risk assessment when using pesticides. This tool serves as a preliminary risk assessment method, offering a simplified model for practical use by farmers. Its initial verification was conducted through a case study and the results represent a step towards promoting safer pesticide practices, providing a solid basis for further validation.

## 1. Introduction

### 1.1. Problem Overview

Farmers’ health is a critical issue in the agricultural sector. A 2012 European Union (EU) survey found that agricultural workers are more likely to report work-related health issues than those in any other sector [1]. Work-related health problems are more prevalent in the agriculture and forestry sector, alongside the mining and quarrying sector, due to unfavorable job conditions such as outdoor activities, the use of heavy vehicles and machinery, and irregular working schedules [2,3,4].

Among the large number of risks that agricultural activities present, pesticide-related ones remain a significant concern [5]. These risks have often been underestimated or inadequately addressed due to a lack of reliable data. Health effects from pesticide exposure range from mild skin and eye irritation to severe outcomes, including cancer and birth defects [6]. These effects may be acute or chronic, and some substances can have cumulative impacts. Certain substances also present safety hazards, such as risks of fire, explosion, or suffocation [7]. Furthermore, effects on the environment, such as the poisoning of water bodies, are strictly related to the improper use of pesticides [8]. A World Health Organization (WHO) and United Nations Environment Programme (UNEP) report highlights that globally three million people are poisoned annually by pesticides, resulting in 200,000 deaths, predominantly in developing countries [9].

In recent years, many countries have implemented legislative frameworks to reduce risks related to the use of pesticides [10]. For example, the European Commission is actively working to reduce pesticide use through Integrated Pest Management (IPM) policy under the Sustainable Use of Pesticides Directive (2009/128/EC) and related action plans. On the one hand, these initiatives are aimed at reducing overall pesticide use by 50% by 2030 and eliminating the most hazardous pesticides. On the other hand, they foster the implementation of safe practices and behaviors by agricultural workers when handling pesticides. However, the complexity of EU legislation on pesticides presents a significant challenge, especially for small farms, as it requires qualified personnel to properly put it into practice [10]. At a general level, current law requirements require specific occupational risk assessment and the use of protective measures for operators dealing with pesticides, but no ad hoc procedures exist to put them into practice [11]. This gap makes it challenging, especially for small-sized companies, to apply a safe procedure to managing pesticides in the workplace [1].

In the foreseeable future, pesticide use in agriculture will persist, and despite the enhanced safety of modern pesticides, ensuring farmers’ protection from unnecessary exposure to these chemicals during all farming activities, particularly during treatments, will remain a priority in most countries, including those outside the European Union [12,13,14].

### 1.2. Pesticide Use Risk Assessment

Risk assessment is at the basis of pesticide workers’ protection and different types of risk assessment can be distinguished in this context: a pre-marketing risk assessment, which consists of a comprehensive risk assessment process for pesticides before they are allowed on the market. A post-marketing risk assessment is conducted during pesticide use for health surveillance of workers who are exposed. Exposure to pesticides in agriculture affects product distributors, mixers and loaders, applicators, bystanders, and rural workers who re-enter fields shortly after treatments. Usually, a risk assessment in real exposure scenarios is conducted through two main tools, that is, environmental and biological monitoring [15,16]. This evaluation is performed ex post, after the exposure has taken place [17].

In addition, an occupational risk assessment activity should also be considered, which consists of the mandatory evaluation of risks the workers are exposed to, which employers have to perform before working activities are carried out. In EU countries, Directive 89/391/EEC requires the issuance of a document that outlines an assessment of safety and health risks at work, including those affecting groups of workers exposed to specific risks. Although in other types of working contexts, such as the manufacturing industry, employers have at their disposal numerous standardized models and tools for carrying out this type of risk assessment, there are not many recognized models for risk assessment when it comes to pesticide use in agriculture [18]. Moreover, the practical application of these OHS provisions requires significant expertise, and challenges may still arise in fully understanding and correctly implementing them [19]. In the Italian context, the agricultural sector is predominantly composed of small-scale or family-run businesses [20,21], where limited human and financial resources often hinder the proper execution of occupational health and safety obligations.

Thus, there is an urgent need in this field for tools able to support companies in properly performing risk assessments related to pesticide use, allowing them, before carrying out the application, to forecast the levels of exposure in a specific working scenario, and thus deciding whether the risk is acceptable or not [8].

### 1.3. Research Background

To address these challenges, the Italian Workers’ Compensation Authority (INAIL) financed a project aimed at the development of a simplified, user-friendly risk assessment procedure, to be implemented in a software tool, to help farmers that use pesticides in meeting OHS requirements effectively.

With this goal in mind, the research started with an analysis of the pesticides’ pre-marketing evaluation. This evaluation is conducted considering the chemical and physical properties of a pesticide to define its Acceptable Operator Exposure Level (AOEL), that is, a limit of exposure defined through a health-based approach in the authorization process, expressed in weight of active ingredient per body weight (mg/kg) [22].

Although risk assessment models based on the AOEL can be reliable tools with which to define the exposure of workers, they can be used in field studies only, as they require laboratory analyses and expert personnel.

In this study, we investigated the possibility of creating a tool that, starting from the experience of the pre-marketing risk assessment, can be used in real-life scenarios, allowing farmers to use the information provided by the pesticide producers in the authorization documents, in combination with data related to the specific application context (e.g., crop height, type of application, type of machinery used, personal protective equipment (PPE) used, etc.), to perform an effective and documented risk assessment in conformity with OHS legislation.

Such a tool must be manageable by any agricultural worker and adequate to perform a preventive risk assessment in most, if not all, the scenarios of exposure present in a defined country.

Since it is already established and accepted that a pesticide application is composed of four main phases (preparation of the mixture to be applied (mixing and loading); pesticide application; re-entry of the worker in the treated field; and post-application cleaning and maintenance), the risk assessment tool should specifically consider these phases.

Overall, the proposed approach was designed to quantitatively and preventively estimate chemical risks from a single pesticide exposure during a workday, under specific usage conditions. The software application, which can be used even by means of mobile phone, allows the prompt calculation of the risk as a percentage of the Acceptable Operator Exposure Limit (AOEL), aiding farmers in planning safe working conditions. Inputs include the pesticide’s phytoiatric efficiency, toxicological hazards, and operational details, such as protective equipment (e.g., gloves, mask, boots, overalls), for tasks like mixture preparation, application, equipment handling, and cleaning.

The developed algorithms to estimate exposure, incorporating factors like the active substance required, agricultural area treated, toxicokinetics (e.g., skin absorption), toxicodynamics (e.g., AOEL), pesticide concentration, etc., are based on established methodologies such as the German Model [23], POEM [24], and EFSA Model [22].

The design and development of a prototype software tool to practically apply such a model was carried out and tested through a case study related to olive cultivation. Due to the specificity of re-entry, this phase was not considered in this prototype.

The remainder of this paper is organized as follows: In Section 2 the general framework of the risk assessment tool is illustrated. Then, Section 3 describes the main parameters that characterize the risk assessment model and their implementation in the Sicurpest software, while in Section 4 the results achieved are discussed. Concluding remarks and further works are reported in Section 5.

## 2. Materials and Methods

The activity that led to the elaboration of the described tool integrates three independent steps:

- Data collection on the typical levels of exposure in different risk and exposure scenarios;
- Definition of the numerical relationships between different determinants of exposure;
- Creation of the model through adequate algorithms.

### 2.1. Data Collection of Typical Levels of Exposure in Different Application Scenarios

In this phase, a literature review was carried out considering major scientific databases (Pubmed, Scopus and Medline) using the search string described by Mattioli et al. [24]. The output of this analysis is summarized in Supplementary Material S1. In particular, 38 articles were selected and used as a base for the definition of the main parameters of risk assessment algorithms to be implemented in the software.

### 2.2. Definition of the Relationships Between Different Determinants of Exposure in the Main Phases of Work with Pesticides

**Figure 1:**
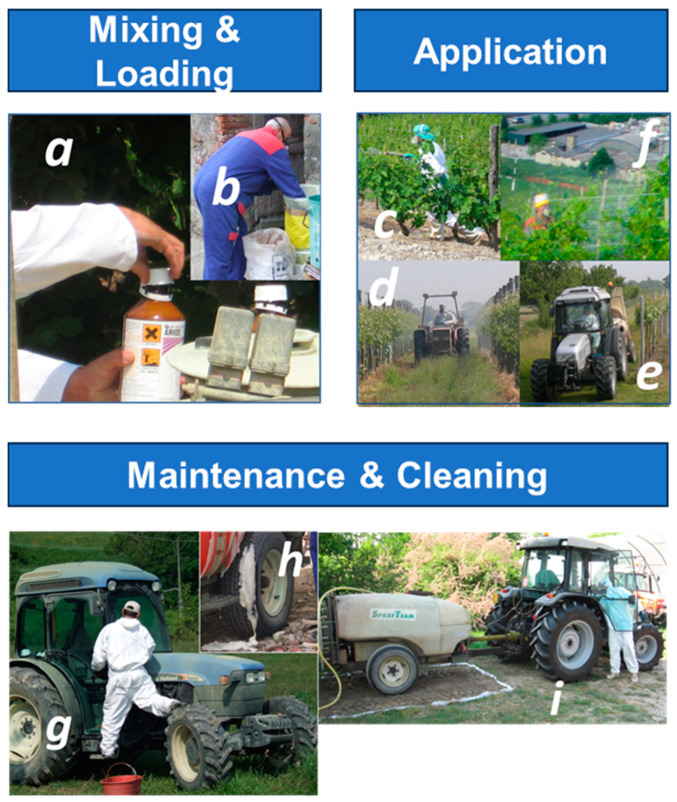
Examples of the main phases of pesticide use. Mixing and loading: concentrated products in powder (**a**) or liquid (**b**) form are handled. Application: different application devices, with a decreasing level of personal contamination, are shown: (**c**) towed pipe; (**d**) spraying from a tractor-towed tank with an open-cockpit tractor; (**e**) a tractor with filtered forced air-sealed cockpit; (**f**) up a steep, terraced hill. Maintenance and cleaning: (**g**) in-field cleaning of cockpit glasses of a tractor with filtered forced air ventilation; (**h**) in-field spillage of product; (**i**) management of pesticide-contaminated washing water by controlled in-soil bio-degradation.

As mentioned above, the agricultural operation of pesticide treatment in the open field encompasses four main phases: (1) mixing and loading; (2) application; and (3) maintenance and cleaning of equipment and non-single-use personal protective devices (Figure 1). The fourth phase, re-entry, has not been addressed at this stage. Each phase is characterized by different exposure determinants, which must be considered for exposure modelling [25].

**Mixing and Loading (M&L)**: this activity includes measuring and dispensing the concentrated product from a commercial bottle or box into the tank, diluting it with the necessary volume of water, and loading it into the spraying tank. In this phase, the operator handles the pesticide concentrate, performs manual operations, and is most prone to being exposed. Liquids can splash or drip from the container to the measuring device, usually a graduated vessel, and from that to the mixing tank.

The main exposure determinants in this phase are the type of formulation (powder, liquid, wettable granules, formulation available in soluble bags), the capacity of the tank, and the number of formulations prepared in a single working day.

The second phase, product **application** (**APP**), is performed in the field and is the phase in which the mixture is applied to the crops. The main exposure determinants to be considered in this phase are the following:

- **Extension** of the treated area;
- **Type of crop**: low and high;
- **Application** modalities: knapsacks (manual and motorized), tractor sprayer, application bar, application pressure;
- **Type of tractor**: open, closed, closed and equipped with charcoal filters.

For the **mixing and loading** and **application** phases, we assumed that the fraction of pesticide reaching the worker depends only on the physical characteristics of the pesticide and the type of application, and not on its chemical composition which, in turn, determines the capacity of penetration of the intact skin and, therefore, pesticide body burden.

The primary goal of our research was to estimate the amount of pesticide mixed and applied that could reach the worker’s skin under various exposure scenarios, in order to assess the relative importance of the different exposure determinants.

**Maintenance and cleaning of equipment (MAN).** This phase includes both in-field interventions and post-application cleaning and maintenance activities. This phase shares with M&L the characteristic of being entirely manual, i.e., consisting of operations that cannot be easily modelized.

The use of PPE differs during the above working activities, as the worker may wear different kinds of PPE depending on the task performed. The most used personal protective devices are hats, generic or specific coveralls, gloves, and boots. Different devices and different materials offer variable degrees of protection. For each type of PPE, various levels of exposure have been calculated through the analysis of studies related to biological and environmental monitoring [26,27,28,29,30,31].

### 2.3. Phytoiatric and Safety Profile of the Pesticides Used in Italy

To carry out the chemical risk assessment process, it is essential to identify the associated hazards. This goal was achieved by gathering information on the properties of active ingredients and related formulations utilized in Italy. The primary data collected for each active ingredient include the usage rate, AOEL, and skin absorption coefficient. In this preliminary version of the tool, only 16 active ingredients have been uploaded, selected based on a priority classification recommended by the Italian Agency for Environmental Protection. This allowed us to create a database containing all parameters related to these 16 products.

### 2.4. Construction of a Physically Based Model and Related Algorithms

**Figure 2:**
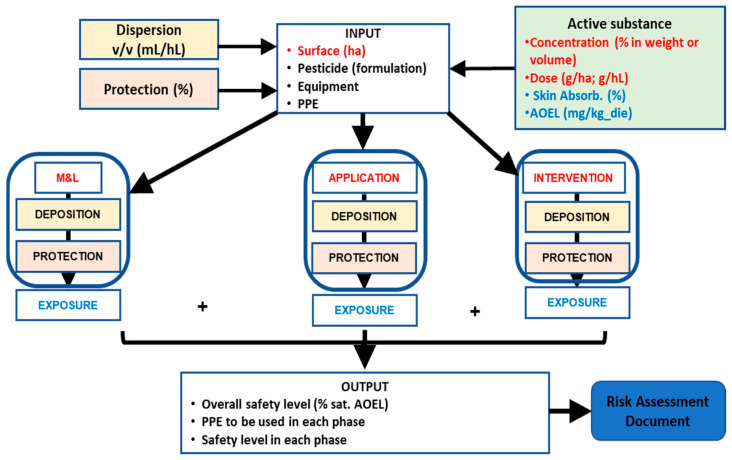
Relational scheme of the main parameters used to develop the risk assessment model.

The development of the risk assessment algorithms follows strict mass-transfer principles that can be modeled with relative ease, using numerical parameter values obtainable from technical sources or calculated through field studies. In contrast, the mixing and loading process can be modeled less rigorously. However, its key exposure characteristics are reasonably predictable, and the extent of exposure is influenced by processes for which parameters can similarly be sourced from technical references or derived from field studies. While in-field and end-of-work manual interventions are less predictable, they share key exposure characteristics with mixing and loading. However, for these activities, exposure parameters can only be estimated using best-guess values from field studies. In Figure 2, the general scheme of the evaluation model is illustrated.

This framework will serve as the foundation for defining the algorithms required to develop the risk assessment model, as described in detail in Supplementary Material S2.

## 3. Results

### 3.1. Risk Assessment Model

Following the framework illustrated in Figure 2, the input data were characterized based on the following information: the surface to be treated, the type of pesticide used, the type of equipment used for pesticide application, and the PPE used. This information is directly linked to deposition and protection parameters. The model considers only one product per time. The main parameters of the pesticide are related to the active substance: concentration, dose, skin absorption, and AOEL. An example of this information is provided in Table 1.

Different products have different potentials of contaminating the operator during mixing and loading. In particular, powders (that can be aero dispersed and remain suspended in air) have the highest chance of contaminating the working environment and the operator, while pre-dosed formulations in soluble bags bring about the lowest levels of exposure. It is also necessary to consider that, in some limited conditions, the M&L phase is not carried out, since the farmer goes to local loading stations where a very limited number of specialists mix the products and load the tanks with automated metering devices. In this case, the estimation of exposure is not performed. In any case, field studies demonstrate that most body exposure can occur in this phase, since the concentrated product is manipulated. This phase can be modelized only considering that a very small volume of the concentrated product comes in contact with the hands and a fraction of that is absorbed. The higher fraction is observed in the handling of compounds in powder form, intermediate in liquid concentrate, and lowest in soluble granules, whilst the use of soluble bags does not involve in this phase any significant exposure to the mixed pesticide. In Table 2 the exposure values depending on the formulation type are shown.

As for the application (APP), the fraction of the product that contaminates the operator depends mainly on the modalities of the spraying operations, with increasing fractions being deposited on the operator who performs the activity manually, such as from a knapsack or by manually towing a spraying pipe. Deposition is lower when the application is directed vertically downwards onto the ground, as in the application of weedkillers in low-lying cultures, or “low crops”, such as cereals. Spray applications on “high crops”, such as of insecticides and fungicides on bush (vineyard) and tree crops, entails higher fractional deposition.

As for the tractor used, an operator inside the driving cockpit of an integrated tractor-tank vehicle with filtered-air ventilation will likely be exposed to a very low fraction of the sprayed product. Of course, it is necessary to consider, with reference to the total amount reaching the worker, that the manual application involves the use of relatively small amounts of pesticide, and the tractor application of high amounts. This will bring about the fact that even if they represent a smaller percentage, the raw amounts of pesticides potentially able to reach the worker involved in tractor application will be higher than those reaching the worker in the manual application, even if the fraction of the pesticide applied is smaller.

The technical modalities of spraying, such as applied air pressure, determine the spray range, droplet size, and the chance of off-target spray drift. In particular, tractor-towed tanks and sprayers range from those with an open driving seat to closed cockpits with a variable degree of isolation, up to a completely sealed cockpit with forced air ventilation and active-charcoal disposable air filters.

The main determinants of exposure in this phase are, therefore, the crop height, with the highest levels of exposure observed in the application on high crops (those in which the worker performs applications directing the flow of the pesticide upwards) and the opposite for low crops, in which the levels of exposure are noticeably lower (in this light, dispensing herbicides on the ground will expose the applicator to a far lower fraction of the product than spraying fungicides and insecticides on trees and vineyards); and the pressure of application, with the toolbar working per gravity providing the lowest levels of exposure compared to other application modalities.

In this phase, it is also necessary to consider the level of protection afforded to the worker by the working clothes. Analogously, an operator equipped with a well-fitting technical coverall will be well protected, while one who wears plain old clothes with generous portions of the body uncovered will be poorly protected. Fractional Protection Factors (PF%) are expressed as the fraction of deposited product that will not pass through and contaminate the operator’s skin. Therefore, very good protection factors, such as those afforded by technical attires, will be close to 95–99%. Of course, the complement to one is the fraction that will pass through and contaminate the operator’s skin.

Table 3 and Table 4 report the values of the Fractional Deposition Coefficients of different modes of pesticide application, as endorsed by the Italian Society for Occupational Medicine and Industrial Hygiene (SIMLII) [32].

In the phase of cleaning and maintenance, most of the time a very small volume of the product comes in contact with the hands and a fraction of that is absorbed. The difference is that, in these operations, the operator comes into contact with the dilute spraying solution, rather than with the concentrated product. This phase is, however, of great concern, since several studies demonstrate that a substantial fraction of the deposited dose comes from contamination of the hands [33,34,35]. Of course, wearing good-quality, well-fitting gloves is the paramount protection measure, especially during operations that most probably entail manual work. However, in several instances, this precaution cannot be effectively practiced, due to the hindrance of technical gloves during fine manual work. In addition, taking gloves off to perform manipulations that lead to hand contamination, and then re-donning the gloves on the contaminated hands, carries additional, even higher pesticide absorption, both in the immediate and on re-use of gloves, the inside of which is already contaminated. Moreover, very often the worker also contaminates the internal parts of the cabin, in particular the steering wheel, the seat, and the control levers of the vehicle, which, in turn, increases the exposure.

Finally, the efficiency of dermal absorption is the determinant that, par being the levels of external dose, and considering the intrinsic toxicity of an active ingredient, identified using the AOEL, determines the occupational risk in pesticide exposure. This parameter is measured with several experimental tools in the toxicological characterization phase of pesticide development or can be estimated with chemometric calculations. Databases compiled by some organizations include the dermal absorption coefficients used for risk assessment in the pre-marketing phase.

The complete set of calculations, with measure units and dimensions, is reported in Supplementary Materials S2.

### 3.2. Description of the Software Tool (Sicurpest, Italian Edition)

This section describes the structure and graphical interface of the IT tool developed in Italian both for smartphone (Android OS) and PC (MS Windows 10) platforms. The structure of the IT tool developed is described here.

For clarity, the information is translated into English.

The first step involves the uploading of the values of the variables in the model.

**Figure 3:**
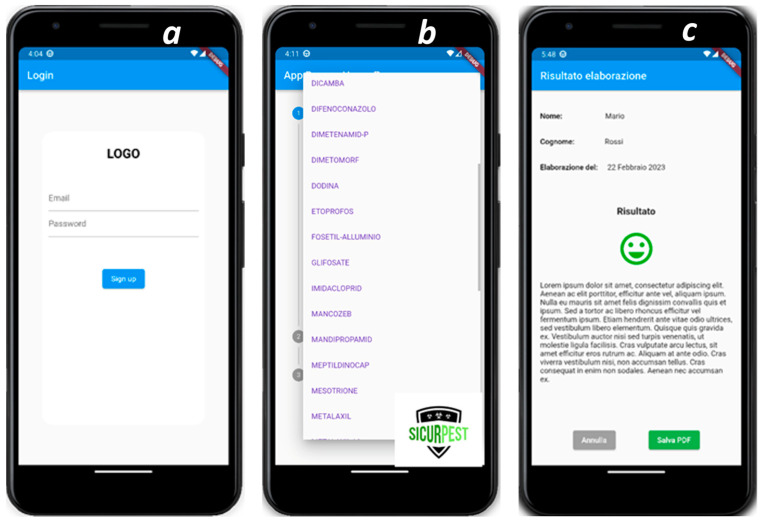
Graphical interface of the smartphone-based Sicurpest app. (**a**) Preliminary registration for the analysis using a username and password; (**b**) representative page of the data entry interface; and (**c**) output of risk assessment (case of a safe procedure).

The identification of variables represents the first phase of preventive risk assessment. Before each application, the worker is asked to perform the following operations: **Log in**: the operator enters their credentials and allows the tool to save the evaluation results on a server, enabling the file to be accessed locally at any time (see Figure 3a).

**Data Entry:** in successive phases, the worker is asked to upload the following data:

- Type of crop;
- Type of pest (Figure 3b);
- Treated surface (ha);
- Active ingredient used (commercial name);
- Physical Form of product (powder; liquid; granules);
- Active ingredient’s concentration.

For each of the three considered working phases, the following information on employed PPEs is collected:

- Face;
- Head;
- Hands;
- Body;
- Feet.

For the application modalities, the following information is requested:

- Volume of applied solution per surface (hL/ha);
- Volume of the tank (liters);
- Spraying pressure (bar);
- Type of spraying equipment (handheld; tractor-different kinds).

**Analysis and result**: the operator views the result of the simulation on the screen, which is also provided in a graphical form (i.e., a “smiling” icon in the case of positive result), and can save the complete analysis report on the server through the report download feature (see Figure 3c).

### 3.3. Application in Olive Culture: Fighting Infestation from the Olive Mosquito Bactrocera Oleae

To verify the correct functioning of the model and software, a case study was carried out considering the risk assessment related to the application of Glyphosate for the treatment of olive trees. This case study was carried out in collaboration with a farmer, who was asked to use the Sicurpest tool by means of a tablet.

The farmer entered all the data regarding the planned activity in Sicurpest and retrieved a quick, manageable risk assessment as a simple three-condition pictogram output: the green “smiling face” for safe application conditions; the yellow “puzzled face” for borderline conditions that may develop into a red “scared face”, indicating unsafe conditions that make application unadvisable and an improvement of working conditions mandatory. At the same time, the farmer can print out the results of the analysis, obtaining a pdf file to be included in the risk assessment document.

The different phases of the use of the tool are schematized in detail in Supplementary Material S3 (Figure S3.1).

In the first phase (Figure S3.1a), the user enters data related to the type of crop, the pest addressed, and the total surface to be treated.

The second module (Figure S3.1b) concerns the “Product”, i.e., the pesticide that should be used. In this version of the tool, the user uploads the chemical name of the active ingredient (AI), the type of formulation (liquid, powder, soluble granules and soluble bags), and the concentration of the AI in the product. All data is reported in the product label or mandatory accompanying documents. Since the exposure coefficients of liquid concentrates and soluble granules are similar, they were merged into a single entry.

The third module (Figure S3.1d) addresses the selection of personal protective equipment (PPE): mask, headdress, specific or generic gloves, body protection (normal dresses or generic/specific coveralls), and feet (shoes or boots). For each type of equipment, it is possible to select “no protection”. This section is repeated for each of the working phases (mixing and loading, application, and cleaning and maintenance), since different PPE can be used and separated calculations are performed.

The fourth module (Figure S3.1e) concerns the volume of the application (hectoliters of solution per hectare), the volume of the employed tank (in Liters), the operating pressure, and the type of equipment used (open and closed tractor; closed and filtered tractor; manual application with boom; and the use of a knapsack, or manual or motorized use).

At the end of the data entry phase, Sicurpest runs the calculation and delivers the risk assessment as an emoji (Figure S3.1f).

Trained users can access the section reporting the numeric values that led to the assessment summarized in the emoji. This numeric section allows the user to understand in which working phase/s the unacceptable risk has been forecasted, thus indicating the needed improvements. The user can then return to the data entry module, change one or more of the critical aspects and run the tool again to verify the improvement.

Once the results are satisfactory, the document can be saved under the name of the employer and attached to the risk assessment documents of the farming company. The pesticide application can only be started when the calculated data will show a situation of acceptable risk.

## 4. Discussion

The current study summarizes the results of a research project financed by the Italian Workers’ Compensation Authority (INAIL) and carried out by the University of Milan and Sapienza University of Rome in Italy in the years 2019–2021.

The project aimed at developing a user-friendly software tool to support farmers in the correct management of pesticide use in agriculture, in compliance with the mandatory occupational health and safety regulations.

In the literature, several examples of risk assessment models for the safe use of pesticides can be found [11,36], but they mostly aimed at evaluating pesticides’ actual risk via post-registration monitoring to minimize the likelihood of future unacceptable risks to human health and the environment [37]. However, few researches have addressed the farmers’ perspective, i.e., the needs of pesticide users to apply these products safely and in a sustainable manner [38]. Accordingly, the current study aimed to reduce this research gap by providing an easy-to-use tool that can foster safe practices among farmers when using pesticides without requiring a high level of expertise.

From the methodological point of view, the knowledge of the relations between different exposure determinants has allowed the creation of a risk assessment model based on the current pesticides databases as the calculation of the risk as a percentage of the Acceptable Operator Exposure Limit (AOEL). This direct link between the practical and contextual situation of the farmer that is going to apply the pesticide on the one hand, and the AOEL values on the other, allows a high level of flexibility in the model (i.e., the risk assessment algorithm), which can be continuously updated based on the latest advancements in the regulatory framework.

The informatization of the model through a prototype software tool provides forecasts of the exposure level to pesticides from the same data that the farmer uses, and allows the user to select the most effective ways to protect themselves from over-exposure, given the availability of agricultural equipment and protective clothing. This preventive decision on pesticide application planning makes use of the information that the Italian farmer, who is usually a trained, registered, and authorized user of pesticides, should routinely keep in the records for inspection by health and safety authorities. The availability and diffusion of this tool may improve the awareness of farmers on the constant use of safe practices in pesticide application. According to the Italian Law for the protection of workers from occupational accidents and diseases, the results of the preventive evaluation can be considered valid as the obligatory risk assessment document for inclusion in the enterprise’s filed documents.

The software tool outlined in this article is a prototype, beta-test version, on which further validation is currently being performed. This activity includes two further parallel verification processes. One is, as expected, an assessment of accuracy, which is achieved through comparing risk assessment calculated by the tool with actual measured exposure and calculated exposure-associated risk. Whenever relevant, calculation equations or the values of crucial parameters can be suitably modified to best match field conditions to the tool model output. Therefore, this version of the Sicurpest tool only includes 16 pesticides with their characteristics, while the INAIL-approved tool will contain a database of all the pesticides authorized in Italy.

The second tier of validation will entail testing for ease-of-use by groups of end-users that include volunteering farmers, for whom the positive “green face” output will be accepted as a formal risk assessment as requested by the Italian law. Another scheduled group will be occupational physician trainees, since, according to Italian law, the occupational physician in charge of the workers’ health surveillance collaborates to carry out risk assessments and develop the risk assessment and prevention plan document of the farm, which is a responsibility of the farm manager. Other interested groups, such as agricultural consultants, trainers of safe pesticide use mandatory courses, and professional pesticide dealers, will be asked for testing support and opinions.

Although working conditions are very variable according to the different nature of the terrain (open flatland to terraced hill), in our own experience in Italy, we never highlighted conditions of unacceptable occupational risk deriving from levels of exposure exceeding the AOEL. In fact, measured exposure was often lower by 1–3 orders of magnitude, even when farmers used the less recent products, endowed with higher toxicity and a lower AOEL value. Therefore, we envisage that the availability of this tool can encourage already complying independent farmers and small-size estates with hired workers to maintain and document their good work.

While this is generally the situation highlighted in studies on agricultural pesticide application in most European EU Countries [33,39,40,41,42,43,44,45,46,47,48,49,50], it should be noted that the most critical situations may escape the observation of research-oriented studies, and even that of regulators’ in-field surveys and inspections. Also in this case, the availability of a quick risk assessment tool for field inspectors can be useful for documenting gross violations of workers’ right to protection.

## 5. Conclusions

Farmer health is a major concern in agriculture, with agricultural workers reporting more work-related health issues than those of any other sector. Pesticide-related risks remain a significant and often underestimated hazard, with potential health effects ranging from mild irritations to severe conditions like cancer and birth defects. These health impacts can be acute or chronic, with some substances having cumulative effects. However, despite the efforts of public authorities to issue an adequate legislative framework to reduce the exposure of farmers, and a large literature analyzing the pesticide products’ features and effects on humans and the environment, the practical needs of farmers to safely manage pesticides are scarcely addressed. The present study presents the outcomes of a project designed to address this research gap by developing software that helps agricultural workers to verify their compliance with mandatory regulations and offers OHS management guidance to establish and maintain safe working conditions during pesticide use.

The development of the Sicurpest tool has addressed several challenges related to the lack of suitable tools for the mandatory risk assessment of agricultural pesticide use in Italy. Upon completion, the strategy behind the tool emphasized making it freely available as an up-to-date, downloadable app, while ensuring its validity within the framework of employer obligations under occupational safety and health laws. This approach benefits both employers and agricultural workers, aligning with the intent of Italian law. This approach mirrors similar self-assessment initiatives in other sectors, part of the EU’s “smart bureaucracy” efforts. The positive results achieved through the development of the Sicurpest prototype, however, require further development. First, additional case studies concerning different cultivations are needed to extend the validity of the tool in the case study context. Then, it must be outlined that the prototype tool was designed for the Italian context only and only the Italian language is available. Hence, further work is needed to make it available at an international level. To achieve this goal, on the one hand, multiple language features are needed, while the adaptation of the pesticide database to other country-specific contexts is required on the other. The authors are currently focused on collaborating with other countries, particularly those in transition or developing regions, where the risk of pesticide exposure may be higher.

## Figures and Tables

**Table 1:** Example of input data related to a pesticide ^1^.

Parameters	Values	Unit	Source
Name	KILLER		Product label
Type	powder		Product label
GAP	100	g/ha_form	Product label
active_subst	ACTIVOL		Product label
Concentration	50	g/100 g	Product label
AOEL	0.05	mg/kg_BW	database
assorb_skin	5	%	database

^1^ Taken from exercise in ref. [32].

**Table 2:** Exposure values depending on the formulation type of the product.

	Exposure	30%	45%	70%	90%
Formulation	Concentration Dispersion (%)	<30%	30–60%	60–80%	>80%
Soluble bags	0%	0%	0%	0%	0%
Liquid concentrate	0.10%	0.030%	0.045%	0.070%	0.090%
Powder	0.01%	0.003%	0.005%	0.007%	0.009%

**Table 3:** Fractional Deposition Coefficients for different modes of pesticide application ^1^.

Clothing Type	Deposition Factor ppm ^2^
Backpack	1000
Towed pipe (manual)	100
Tractor (open cockpit) with towed tank	10
Tractor (closed cockpit, natural ventilation) with towed tank	1
Tractor (closed cockpit, forced ventilation) with towed tank	0.1
Tractor (closed cockpit, forced ventilation, charcoal-filtered air) with towed tank	99%

^1^ The information in this table merges data reported from different, heterogeneous sources [32]; ^2^ parts-per-million = 0.1 mL/hL or mg/kg.

**Table 4:** Fractional Protection Coefficients for different clothing worn during pesticide application ^1^.

Clothing Type	Protection Factor ^2^
Underwear	50%
Generic clothes	70%
Cotton coverall (reusable)	90%
Non-woven fabric single-use coverall	90%
HazMat disposable coverall	99%

^1^ The information in this table merges data reported from different, heterogeneous sources [32]; ^2^ fraction that does not cross the barrier.

## Data Availability

The raw data of the monitoring studies are published in the referenced literature. Details of the models are disclosed in this article or in the referenced literature. The software code is not available, as the funder retains exclusive rights to this product.

## Supplementary Materials

The following supporting information can be downloaded at: https://www.mdpi.com/article/10.3390/toxics13020089/s1, S1: Supplementary reference list; S2: Calculation scheme; S3: Example of tool application.
