# Supplementary material for: Sicurpest: A Prototype of a User-Friendly Tool for Preventive Risk Assessment of Pesticide Use in Agriculture
Source: Toxics. 2025 Jan 24;13(2):89. doi: 10.3390/toxics13020089 (PMC11861541; doi:10.3390/toxics13020089)
Supplement: Supplementary file 1 [file toxics-13-00089-s001.zip › toxics-3417385-supplementary.pdf]

toxics

MDPI

Federico Maria Rubino, Daniele Puri, Mario Fargnoli, Mara Lombardi, Stefan Mandic-Rajcevic and Claudio Colosio. **Sicurpest: a prototype of a user-friendly tool for preventive risk assessment of pesticide use in agriculture. Toxics (2025) doi:**

Supplementary Information S1

Selected articles (merged Pubmed and Scopus databases)

[1] 3. Regul Toxicol Pharmacol. 2020 Aug;115:104689. doi: 10.1016/j.yrtph.2020.104689. Epub 2020 Jun 13. Establishing health-based biological exposure limits for pesticides: A proof of principle study using mancozeb. Mandić-Rajčević S(1), Rubino FM(2), Colosio C(2). Author information: (1)Department of Health Sciences of the University of Milan and International Centre for Rural Health of the San Paolo Hospital, Via San Vigilio 43, 20142, Milan, Italy. Electronic address: stefan.mandic-rajcevic@unimi.it. (2)Department of Health Sciences of the University of Milan and International Centre for Rural Health of the San Paolo Hospital, Via San Vigilio 43, 20142, Milan, Italy. Pesticides represent an economical, labor-saving, and efficient tool for pest management, but their intrinsic toxic properties may endanger workers and the general population. Risk assessment is necessary, and biological monitoring represents a potentially valuable tool. Several international agencies propose biological exposure indices (BEI), especially for substances which are commonly absorbed through the skin. Biological monitoring for pesticide exposure and risk assessment seems a natural choice, but biological exposure limits (BEL) for pesticides are lacking. This study aims at establishing equivalent biological exposure limits (EBEL) for pesticides using real-life field data and the Acceptable Operator Exposure Level (AOEL) of mancozeb as the reference. This study included a group of 16 vineyard pesticide applicators from Northern Italy, a subgroup of a more extensive study of 28 applicators. Their exposure was estimated using "patch" and "hand-wash" methodologies, together with biological monitoring of free ethylene-bis-thiourea (ETU) excretion in 24-h pre- and post-exposure urine samples. Modeling was done using univariate linear regression with ETU excretion as the dependent variable and the estimated absorbed dose as the independent variable. The median skin deposition of mancozeb in our study population was 125 µg, leading to a median absorbed dose of 0.9 µg/kg. The median post-exposure ETU excretion was 3.7 µg. The modeled EBEL for mancozeb was 148 µg of free ETU or 697 µg of total ETU, accounting for around 75% of the maximum theoretical excretion based on a mass balance model. Although preliminary and based on a small population of low-exposed workers, our results demonstrate a procedure to develop strongly needed biological exposure limits for pesticides. Copyright © 2020 Elsevier Inc. All rights reserved. DOI: 10.1016/j.yrtph.2020.104689 PMID: 32544413 [Indexed for MEDLINE] Conflict of interest statement: Declaration of competing interest The authors declare that they have no known competing financial interests or personal relationships that could have appeared to influence the work reported in this paper.

[2] Environ Sci Pollut Res Int. 2019 Jan;26(2):1642-1653. doi: 10.1007/s11356-018-3676-5. Epub 2018 Nov 17. Assessment of occupational exposure to pesticide mixtures with endocrine-disrupting activity. Wong HL(1)(2), Garthwaite DG(3), Ramwell CT(3), Brown CD(4). Author information: (1)Environment Department, University of York, York, YO10 5NG, UK. hw1166@york.ac.uk. (2)Faculty of Earth Science, University Malaysia Kelantan, Locked Bag 100, 17600, Jeli, Kelantan, Malaysia. hw1166@york.ac.uk. (3)Fera Science Ltd. (Fera), Sand Hutton, York, YO41 1LZ, UK. (4)Environment Department, University of York, York, YO10 5NG, UK. Occupational exposure to pesticide mixtures comprising active substance(s) and/or co-formulant(s) with known/possible endocrine-disrupting activity was assessed using long-term activity records for 50 professional operators representing arable and orchard cropping systems in Greece, Lithuania, and the UK. Exposure was estimated using the harmonised Agricultural Operator Exposure Model, and risk was quantified as a point of departure index (PODI) using the lowest no observed (adverse) effect level. Use of substances with known/possible endocrine activity was common, with 43 of the 50 operators applying at least one such active substance on more than 50% of spray days; at maximum,

one UK operator sprayed five such active substances and 10 such co-formulants in a single day. At 95th percentile, total exposure was largest in the UK orchard system (0.041 × 10-2 mg kg bw-1 day-1) whereas risk was largest in the Greek cropping systems (PODI 0.053 × 10-1). All five cropping systems had instances indicating potential for risk when expressed at a daily resolution (maximum PODI 1.2-10.7). Toxicological data are sparse for co-formulants, so combined risk from complex mixtures of active substances and co-formulants may be larger in reality. DOI: 10.1007/s11356-018-3676-5 PMID: 30448946 [Indexed for MEDLINE]

[3] 22. Ann Work Expo Health. 2018 Oct 15;62(8):1040-1046. doi: 10.1093/annweh/wxy056. Physiological Strain in French Vineyard Workers Wearing Protective Equipment to Conduct Re-Entry Tasks in Humid Conditions. Grimbuhler S(1), Viel JF(2)(3). Author information: (1)IRSTEA, National Research Institute of Science and Technology for Environment and Agriculture, Research team "Information - Technologies - Environmental analysis - Agricultural processes", 361 rue Jean-François Breton, Montpellier, France. (2)Univ Rennes, CHU Rennes, Inserm, EHESP, Irset (Institut de recherche en santé, environnement et travail) - UMR_S 1085, 9 avenue du Professeur Léon Bernard, Rennes, France. (3)Department of Epidemiology and Public Health, University Hospital, 2 rue Henri le Guilloux, Rennes, France. The proper use of personal protective equipment (PPE) plays an important role in reducing exposure to pesticides in vineyard farming activities, including re-entry tasks. However, discomfort from clothing systems may increase the physiological burden on workers. We compared the physiological burdens of vineyard workers wearing three different types of PPE during canopy management in field humid conditions while accounting for occupational, climatic, and geographical environments. The study was conducted in the Bordeaux vineyards of southern France during June 2012. A total of 42 workers from seven vineyards consented to field observations. The following PPE garments were randomly allocated: HF Estufa polyamide (Brisa®), Tyvek® Classic Plus, and Tychem® C Standard. Participant sociodemographic characteristics were collected using a structured questionnaire. Skin temperature and heart rate were monitored continuously using portable devices. Multivariate multilevel linear regression models were performed to account for the hierarchical structure of data. No significant difference was found for mean skin temperature during work. Regardless of the cardiac strain parameter considered, the Tyvek® Classic Plus garment produced the poorest results ($P \leq 0.03$). Under the very humid conditions encountered during the field study, the thinness and breathability of the Tyvek® Classic Plus garment resulted in undergarment humidity, imposing additional physiological burden on vineyard workers. These results confirm that the idea of using generic coveralls in any farming activity is unsuitable. Compromises should be created between physiological costs and protection, depending on the agricultural task performed, the crop grown, and the environmental conditions encountered. DOI: 10.1093/annweh/wxy056 PMID: 29924310 [Indexed for MEDLINE]

[4] J Prev Med Hyg. 2018 Sep 28;59(3):E200-E211. doi: 10.15167/2421-4248/jpmh2018.59.3.934. eCollection 2018 Sep. Health and safety of pesticide applicators in a high income agricultural setting: a knowledge, attitude, practice, and toxicity study from North-Eastern Italy. Riccò M(1)(2), Vezzosi L(3), Gualerzi G(4). Author information: (1)Provincial Agency for Health Services of the Autonomous Province of Trento, Department of Prevention, Occupational Health and Safety Unit (UOPSAL), Trento (TN), Italy. (2)Azienda USL-IRCCS di Reggio Emilia, Dipartimento di Sanità Pubblica, Servizio di Prevenzione e Sicurezza degli Ambienti di lavoro, Reggio Emilia, Italy. (3)University of Campania "Luigi Vanvitelli", Department of Experimental Medicine, Naples (NA), Italy. (4)University of Parma, Department Medicine and Surgery, School of Medicine and Surgery, Parma (PR), Italy. BACKGROUND: We assessed knowledge, attitudes and practices regarding pesticide handling and related health problems among pesticide applicators (PAs) from the Autonomous Province of Trento, Italy. METHODS: A cross-sectional questionnaire-based study was performed in spring 2016, involving 260 PAs. Logistic regression analyses were used to identify factors associated with a safer use of pesticides. RESULTS: The mean age of participants was 48.8 ± 13.2 years. 89.2% were males. Use of personal protective equipment (PPE) was diffuse, particularly gloves (92.7%), face mask (91.2%), and post-spraying personal hygiene practices were extensively applied. Overall, 43.5% had experienced pesticide-related symptoms especially in subjects misusing PPE, but also for avoiding hygienic procedures. Knowledge about pesticides was a

significant predictor for frequency of symptoms. CONCLUSIONS: As a better knowledge of pesticide-related risks was a significant predictor to reduce symptoms, our results stress that improving awareness and promoting safe use of pesticide may improve the health of PAs. DOI: 10.15167/2421-4248/jpmh2018.59.3.934 PMCID: PMC6196375 PMID: 30397676 [Indexed for MEDLINE] Conflict of interest statement: Conflict of interest statement None declared.

[5] Swiss Med Wkly. 2018 Apr 26;148:w14610. doi: 10.4414/smw.2018.14610. eCollection 2018. Occupational exposure to plant protection products and health effects in Switzerland: what do we know and what do we need to do? Graczyk H(1), Hopf NB(1), Mediouni Z(1), Guseva-Canu I(1), Sanvido O(2), Schmid K(2), Berthet A(1). Author information: (1)Institute for Work and Health, University of Lausanne, Epalinges-Lausanne, Switzerland. (2)State Secretariat for Economic Affairs (SECO), Bern, Switzerland. AIMS: There is currently no centralised database on workers' exposures to plant protection products (PPPs) in Switzerland, nor a national register for negative health effects linking them to occupational PPP exposure. This lack of basic data makes it difficult to implement either epidemiological research or prevention campaigns for the agricultural sector. The first objective was to understand the level of information and flow of data on occupational PPP exposures and health effects in the Canton of Vaud, Switzerland. Then, to apply this information to develop recommendations for improving a vigilance system for occupational health effects related to PPP exposure. METHODS: A mapping study and semistructured stakeholder interviews were conducted to better understand the flow of data on occupational PPP exposures and health effects. A clinical records investigation of workers occupationally exposed to PPPs was undertaken to understand the magnitude of this potential problem. Finally, a workshop brought together relevant stakeholders to discuss recommendations for the way forwards. RESULTS: A lack of data on PPP exposures and associated health effects was revealed. This highlighted important knowledge gaps at different levels of the current institutional information flow system. We found that although there were numerous stakeholders that worked efficiently in their own mandate, there was a clear need for increased collaboration and coordination in order to make use of existing data to promote safer PPP use among agricultural workers in Switzerland. CONCLUSIONS: Due to increasing evidence of an association between PPP exposure and health effects, increased collaboration between stakeholders is necessary to develop links between the data sources that already exist. Our study was the first to investigate the health effects linked to PPP exposure among the Swiss agricultural population. The recommendations presented in this paper would help promote a safer and healthier agricultural workforce in Switzerland, as well as the population at large. DOI: 10.4414/smw.2018.14610 PMID: 29698541 [Indexed for MEDLINE]

[6] Sci Total Environ. 2018 Apr 1;619-620:874-882. doi: 10.1016/j.scitotenv.2017.11.127. Epub 2017 Nov 29. Assessment of exposure of professional agricultural operators to pesticides. Wong HL(1), Garthwaite DG(2), Ramwell CT(2), Brown CD(3). Author information: (1)Environment Department, University of York, York YO10 5NG, United Kingdom; Faculty of Earth Science, University Malaysia Kelantan, Locked Bag 100, Jeli 17600, Kelantan, Malaysia. Electronic address: hw1166@york.ac.uk. (2)Fera Science Ltd (Fera), Sand Hutton, York YO41 1LZ, United Kingdom. (3)Environment Department, University of York, York YO10 5NG, United Kingdom. This study investigates how field practices in handling and applying pesticides influence the long-term patterns of professional agricultural operators' exposure to pesticides. It presents the first use of a comprehensive pesticide application dataset collected on behalf of the European Food Safety Authority with 50 operators selected to cover arable and orchard cropping systems in Greece, Lithuania and the UK. Exposure was predicted based on the harmonised Agricultural Operator Exposure Model (AOEM) and compared with Acceptable Operator Exposure Levels (AOELs). The amount of pesticides handled by individual operators across a cropping season was largest in the UK arable and orchard systems (median 580 and 437kg active substance, respectively), intermediate for the arable systems in Greece and Lithuania (151 and 77kg, respectively), and smallest in the Greek orchard system (22kg). Overall, 30 of the 50 operators made at least one application within a day with predicted exposure greater than the AOEL. The rate of AOEL exceedance was greatest in the Greek cropping systems (8 orchard operators, 2.8-16% of total applications; 7 arable operators, 1.1-14% of total applications), and least for the Lithuanian arable system (2 operators, 2.9-4.5% of total

applications). Instances in Greece when predicted exposure exceed the AOEL were strongly influenced by the widespread use of wettable powder formulations (>40% of the total pesticide active substance handled for 11 of the 20 Greek operators). In contrast, the total area of land treated with an active substance on a single day was more important in the UK and Lithuania (95th percentile observed value was 132 and 19haday-1 for UK arable and orchard systems, respectively). Study findings can be used to evaluate current assumptions in regulatory exposure calculations and to identify situations with potential risk that require further analysis including measurements of exposure to validate model estimations. Copyright © 2017 Elsevier B.V. All rights reserved. DOI: 10.1016/j.scitotenv.2017.11.127 PMID: 29734633 [Indexed for MEDLINE]

[7] Environmental and biological monitoring for the identification of main exposure determinants in vineyard mancozeb applicators. Mandic-Rajcevic S(1), Rubino FM(1), Ariano E(2), Cottica D(3), Neri S(3), Colosio C(1). Author information: (1)Department of Health Sciences of the University of Milan and International Centre for Rural Health of the San Paolo Hospital, Via San Vigilio 43, 20142 Milan, Italy. (2)Working Group for Prevention in Agriculture of the Region of Lombardy, Milan, Italy. (3)Centre for Environmental Research, Fondazione Salvatore Maugeri, Pavia, Italy. Grapevine is a vulnerable crop to several fungal diseases often requiring the use of ethylenebisdithiocarbamate (EBDC) fungicides, such as mancozeb. This fungicide has been reported to have goitrogenic, endocrine disrupting, and possibly immunotoxic effects. The aim of this study was to assess workers' exposure in two scenarios of mancozeb application and analyse the main determinants of exposure in order to better understand their mechanism of influence. Environmental monitoring was performed using a modified Organisation for Economic Co-operation and Development (OECD) "patch" methodology and by hand-wash collection, while mancozeb's metabolite, ethylenethiourea (ETU), was measured in 24-h preexposure and postexposure urine samples. Liquid chromatography-mass spectrometry was used for determination of mancozeb and ETU in different kinds of samples. Closed tractor use resulted in 40 times lower potential exposure compared with open tractor. Coveralls reduced skin exposure 4 and 10 times in case of open and closed tractors, respectively. Gloves used during application resulted in 10 times lower hand exposure in open but increased exposure in closed tractors. This study has demonstrated that exposure to mancozeb is low if safe occupational hygiene procedures are adopted. ETU is confirmed as suitable biological marker of occupational exposure to mancozeb, but the absence of biological exposure limits significantly reduces the possibility to interpret biological monitoring results in occupationally exposed workers. DOI: 10.1038/jes.2017.14 PMID: 28901326 [Indexed for MEDLINE]

[8] Med Tr Prom Ekol. 2017;(2):1-6. Insecticides of neonicotinoides class: determining exposure via workers' urine. [Article in English, Russian] Rakitskiy VN, Fedorova NE, Bayusheva VV, Chistova ZA. For biomonitoring of exposure in workers with insectocaricides, the authors created a method of multi-component assessment oflowlevels of neonicotinomides in workers' urine, based on last generation tandem liquid mass-spectrometry (triple quadrupole) with ionization source - electrostatic dispersion (positive ionization) in dynamic multi-reaction monitoring with two transitions of parent ions (for quantitative assessment and ionic ratio confirmation). After the work, the operators gave urine samples (about 100 ml in average) that were frozen and kept under -20ºC before analysis. Samples were defrozen before analysis, and each urine portion of 5 ml was diluted by equal volume of 0,1% formic acid. To extract substances out of the samples, solid-phase extraction (cartridges based on octadecylsilane) was applied, elution was performed with 1 ml of methanol. Lower limit of the substances detection in urine - 0,02-0,05 ng/ml, lower limit of the quantitative assessment - 0,1-0,2 ng/ml. The method was tested on monitoring of the workers' exposure to preparations based on imidaclopride and clotianidine in natural conditions of pesticides use in agriculture with various processing technologies. midacloprid was identified in urine of 3 professional operators after wheat and oat seeds treatment and after subsequent seeding at lower limit of detection (0,02 ng/ml), lower limit of quantitative assessment (0,1 ng/ml) and 0,34 ng/ml. PMID: 30351839 [Indexed for MEDLINE]

[9] Int J Occup Saf Ergon. 2017 Jun;23(2):229-239. doi: 10.1080/10803548.2016.1195130. Epub 2016 Jul 6. Operator dermal exposure and protection provided by personal protective equipment and working coveralls during mixing/loading, application

and sprayer cleaning in vineyards. Thouvenin I(1), Bouneb F(2), Mercier T(2). Author information: (1)a HumExpo SA , France. (2)b French Agency for Food , France. The efficiency of a working coverall combined with personal protective equipment to protect operators against dermal exposure to plant protection products under field conditions was studied. Operators wore a non-certified water-repellent finish polyester/cotton coverall plus a certified gown during the mixing/loading and the cleaning phases. Insecticide foliar application to a vineyard was selected as the exposure scenario. The overall dermal residue levels measured in this study were in the range of data recently collected in Europe. The water-repellent finish working coverall reduced body exposure by a factor of approximately 95%. Wearing a Category III Type 3 partial body gown during mixing/loading and cleaning of the application equipment led to a further protective effect of 98.7%. The combination of a water-repellent finish working coverall and partial body protection during specific tasks provided satisfactory levels of protection and can be considered as suitable protection for the conditions of use studied. DOI: 10.1080/10803548.2016.1195130 PMID: 27384244 [Indexed for MEDLINE]

[10] J Occup Environ Hyg. 2016;13(6):476-89. doi: 10.1080/15459624.2016.1143948. Assessment of exposure to pesticides during mixing/loading and spraying of tomatoes in the open field. Aprea MC(1), Bosi A(2), Manara M(2), Mazzocchi B(2), Pompini A(2), Sormani F(2), Lunghini L(1), Sciarra G(1). Author information: (1)a Public Health Laboratory, National Health Service , Siena , Italy. (2)b Operative Unit Prevention and Safe in Working Place, Public Health Department, National Health Service Piacenza , Italy. Some evidence of exposure-response of metolachlor and pendimethalin for lung cancer and an association of metribuzin with risk of glioma have been reported. The primary objectives in this study were to evaluate exposure and occupational risk during mixing/loading of pesticides and during their application to tomatoes cultivated in open fields. Sixteen farmers were sampled. Respiratory exposure was estimated by personal air sampling using fiberglass filters in a IOM device. Dermal exposure was assessed using skin pads and hand washing. Absorbed doses were estimated assuming 100% lung retention, and 50% or 10% skin absorption for metribuzin, and pendimethalin and metolachlor, respectively. The three pesticides were quantified by gas chromatography tandem mass spectrometry in all matrices. Metolachlor was used as a tracer of contamination of clothes and tractors unrelated to the exposure monitored. Respiratory exposure to metribuzin, used in granular form, was on average more than one order of magnitude higher than exposure to pendimethalin, used in the form of microencapsulated liquid. The actual doses were 0.067-8.08 µg/kg bw, 0.420-12.6 µg/kg bw, and 0.003-0.877 µg/kg bw for pendimethalin, metribuzin, and metolachlor, respectively. Dermal exposure was about 88% of the actual dose for metribuzin and more than 95%, for pendimethalin and metolachlor. For risk assessment, the total absorbed doses (sum of respiratory and skin absorbed doses) were compared with the AOEL for each compound. The actual and absorbed doses of the three pesticides were always lower than the acceptable operator exposure level (AOEL), which are reported to be 234 µg/kg bw, 20 µg/kg bw, and 150 µg/kg bw for pendimethalin, metribuzin, and metolachlor, respectively. In any case, personal protective equipment and spraying devices should be chosen with care to minimize exposure. DOI: 10.1080/15459624.2016.1143948 PMID: 26853603 [Indexed for MEDLINE]

[11] Med Lav. 2015 Jul 8;106(4):294-315. Dermal exposure and risk assessment of tebuconazole applicators in vineyards. Mandic-Rajcevic S(1), Rubino FM, Vianello G, Fugnoli L, Polledri E, Mercadante R, Moretto A, Fustinoni S, Colosio C. Author information: (1)Department of Health Sciences of the University of Milan, International Centre for Rural Health of the San Paolo Hospital and Laboratory for Analytical Toxicology and Metabolomics (LaTMA). stefan.mandicrajcevic@gmail.com. INTRODUCTION: Models used in the pre-marketing evaluation do not cover all work scenarios and may over- or underestimate exposure. OBJECTIVES: Uncertainties present in the extrapolation from pre-marketing to the post-marketing warrant exposure and risk assessment in real-life working conditions. METHODS: Seven vineyard pesticide applicators were followed for a total of 12 work-days. A data collection sheet was developed specifically for this study. Workers' body exposure, hands, and head exposure were measured. Tebuconazole was analyzed using LC-MS/MS. RESULTS: Median potential and actual body exposures were 22.41 mg/kg and 0.49 mg/kg of active substance applied, respectively. The median protection

factor provided by the coverall was 98% (range: 90-99%). Hand exposure was responsible for 61% of total actual exposure, and was reduced by more than 50% in workers using gloves. The German Model underestimated the exposure in one work-day, and grossly overestimated it in 3 work-days. CONCLUSIONS: High levels of potential body exposure were efficiently controlled by the cotton coverall. Use of personal protective devices, especially chemically-resistant gloves and head cover is the main determinant of skin protection. Field studies on pesticide exposure in real-life conditions and development of methods and tools for easier risk assessment are necessary to complement and confirm the risk assessment done in the authorization process. PMID: 26154472 [Indexed for MEDLINE]

[12] Food Chem Toxicol. 2015 May;79:54-64. doi: 10.1016/j.fct.2015.02.008. New approaches to uncertainty analysis for use in aggregate and cumulative risk assessment of pesticides. Kennedy MC(1), van der Voet H(2), Roelofs VJ(3), Roelofs W(3), Glass CR(3), de Boer WJ(2), Kruisselbrink JW(2), Hart AD(3). Author information: (1)The Food and Environment Research Agency (Fera), Sand Hutton, York, UK. Electronic address: marc.kennedy@fera.gsi.gov.uk. (2)Biometris, Wageningen University and Research Centre, Wageningen, The Netherlands. (3)The Food and Environment Research Agency (Fera), Sand Hutton, York, UK. Risk assessments for human exposures to plant protection products (PPPs) have traditionally focussed on single routes of exposure and single compounds. Extensions to estimate aggregate (multi-source) and cumulative (multi-compound) exposure from PPPs present many new challenges and additional uncertainties that should be addressed as part of risk analysis and decision-making. A general approach is outlined for identifying and classifying the relevant uncertainties and variabilities. The implementation of uncertainty analysis within the MCRA software, developed as part of the EU-funded ACROPOLIS project to address some of these uncertainties, is demonstrated. An example is presented for dietary and non-dietary exposures to the triazole class of compounds. This demonstrates the chaining of models, linking variability and uncertainty generated from an external model for bystander exposure with variability and uncertainty in MCRA dietary exposure assessments. A new method is also presented for combining pesticide usage survey information with limited residue monitoring data, to address non-detect uncertainty. The results show that incorporating usage information reduces uncertainty in parameters of the residue distribution but that in this case quantifying uncertainty is not a priority, at least for UK grown crops. A general discussion of alternative approaches to treat uncertainty, either quantitatively or qualitatively, is included. DOI: 10.1016/j.fct.2015.02.008 PMID: 25688423 [Indexed for MEDLINE]

[13] 76. Food Chem Toxicol. 2015 May;79:45-53. doi: 10.1016/j.fct.2014.12.012. Testing a cumulative and aggregate exposure model using biomonitoring studies and dietary records for Italian vineyard spray operators. Kennedy MC(1), Glass CR(2), Fustinoni S(3), Moretto A(4), Mandic-Rajcevic S(5), Riso P(6), Turrini A(7), van der Voet H(8), Hetmanski MT(2), Fussell RJ(2), van Klaveren JD(9). Author information: (1)The Food and Environment Research Agency (Fera), Sand Hutton, York, UK. Electronic address: marc.kennedy@fera.gsi.gov.uk. (2)The Food and Environment Research Agency (Fera), Sand Hutton, York, UK. (3)Department of Clinical Sciences and Community Health, University of Milano and Fondazione IRCCS Ca' Granda Ospedale Maggiore Policlinico, Milan, Italy. (4)Department of Biomedical and Clinical Sciences, University of Milan, Milan, Italy. (5)Department of Health Sciences of the University of Milan, San Paolo Hospital Unit, and International Centre for Rural Health of the San Paolo Hospital, Milan, Italy. (6)Department of Food, Environmental and Nutritional Sciences, University of Milan, Milan, Italy. (7)Consiglio per la Ricerca e la sperimentazione in Agricoltura (CRA)- Centro di Ricerca per gli Alimenti e la NUTrizione (CRA-NUT), Via Ardeatina 546, Rome I-00178, Italy. (8)Biometris, Wageningen University and Research Centre, Wageningen, The Netherlands. (9)National Institute for Public Health and the Environment (RIVM), Bilthoven, The Netherlands. The need for improved tools to estimate the cumulative and aggregate exposure to compounds such as plant protection products (PPPs) is recognised in the EU Regulation 1107/2009. A new model has been developed to estimate the exposure within a population to single compounds or compounds within a Cumulative Action Group, considering dietary and non-dietary sources and multiple exposure routes. To test the model a field study was carried out in Italy with operators applying tebuconazole fungicides, with measurements of dermal exposure collected. Whole urine samples were

collected and analysed to provide values for the absorbed dose of tebuconazole, with duplicate diet samples collected and analysed as a measure of dietary exposures. The model provided predicted values of exposure for combined dietary and non-dietary routes of exposures which were compared to the measured absorbed dose values based on urinary analysis. The model outputs provided mean daily exposure values of 1.77 (± 1.96) µg a.s./kg BW which are comparable to measured mean values from the biomonitoring field study of 1.73 (± 1.31) µg a.s./kg BW. To supplement the limited measurement data available, comparisons against other models were also made and found to be comparable. DOI: 10.1016/j.fct.2014.12.012 PMID: 25542526 [Indexed for MEDLINE]

[14] Sci Total Environ. 2015 Feb 1;505:1082-92. doi: 10.1016/j.scitotenv.2014.10.099. Perceptions of pesticides exposure risks by operators, workers, residents and bystanders in Greece, Italy and the UK. Remoundou K(1), Brennan M(1), Sacchettini G(2), Panzone L(1), Butler-Ellis MC(3), Capri E(2), Charistou A(4), Chaideftou E(4), Gerritsen-Ebben MG(5), Machera K(4), Spanoghe P(6), Glass R(7), Marchis A(2), Doanngoc K(6), Hart A(7), Frewer LJ(8). Author information: (1)Food and Society Group, Centre for Rural Economy, School of Agriculture, Food and Rural Development, Newcastle University, Newcastle Upon Tyne NE19 1AA, UK. (2)Opera Research Centre, Università Cattolica del Sacro Cuore di Piacenza, 29100 Piacenza, Italy. (3)Silsoe Spray Applications Unit, NIAB, Building 42, Wrest Park, Silsoe, Bedford MK45 4HP, UK. (4)Benaki Phytopathological Institute, 8 Stefanou Delta Street, Kifissia, Athens 14561, Greece. (5)TNO Innovation for Life, Utrechtseweg 48, 3704 HE Zeist, The Netherlands. (6)Department of Crop Protection, Ghent University, Coupure Links 653, B-9000 Ghent, Belgium. (7)Food and Environmental Research Agency, Sand Hutton, York Y0411LZ, UK. (8)Food and Society Group, Centre for Rural Economy, School of Agriculture, Food and Rural Development, Newcastle University, Newcastle Upon Tyne NE19 1AA, UK. Electronic address: Lynn.Frewer@newcastle.ac.uk. The EU Directive on the sustainable use of pesticides (EU128/2009/EC) requires European Member States to develop training activities targeting occupational exposure to pesticides, and communication material aimed at residents and bystanders. Risk perceptions, knowledge and attitudes associated with passive and occupational exposure to pesticide potentially influence the extent to which different stakeholders adopt self-protective behaviour. A methodology for assessing the link between attitudes, adoption of self-protective behaviours and exposure was developed and tested. A survey was implemented in the Greece, Italy and the UK, and targeted stakeholders associated with pesticide exposure linked to orchards, greenhouse crops and arable crops respectively. The results indicated that the adoption of protective measures is low for residents and bystanders, with the exception of residents in Greece, when compared to operators and workers, who tend to follow recommended safety practices. A regression analysis was used to examine the factors affecting the probability of adopting protective measures as well the as the level of exposure in the case of operators and workers where data are available. The results indicate that the likelihood of engaging in self-protective behaviour is not significantly affected by perceptions of own health being affected by pesticides for residents and bystanders. However, operators who perceive that their heath has been negatively affected by the use of pesticides are found to be more likely to adopt self-protective behaviours. Gender and country differences, in perceptions, attitudes and self-protection are also observed. Recommendations for improved communication, in particular for vulnerable groups, are provided. DOI: 10.1016/j.scitotenv.2014.10.099 PMID: 25461109 [Indexed for MEDLINE]

[15] 79. Chem Res Toxicol. 2014 Nov 17;27(11):1943-9. doi: 10.1021/tx500291t. Identification and quantification of metabolites of the fungicide tebuconazole in human urine. Mercadante R(1), Polledri E, Scurati S, Moretto A, Fustinoni S. Author information: (1)Department of Clinical Sciences and Community Health, Università degli Studi di Milano and Fondazione IRCCS Ca' Granda Ospedale Maggiore Policlinico , Milan, Italy. Tebuconazole (TEB) is a fungicide used in agriculture; the objective of this work was to identify and quantify TEB metabolites in human urine. Samples from seven vineyard workers exposed to TEB were submitted to liquid chromatography interfaced with a triple quadrupole mass spectrometer, equipped with an electron spray source, and a linear ion trap to gain a profile of candidate metabolites. Based on the presence of the ion m/z 70 in the MS/MS spectra, which corresponds to protonated triazole (a specific moiety of TEB), and the isotopic pattern of

the molecular ions, typical of molecules with one chlorine atom, hydroxyl and carboxyl derivatives of TEB, that is, TEB-OH and TEB-COOH, were identified as major metabolites, both as free molecules and as glucuronide (Glc) conjugates. The mean molar fractions were 0.67, 0.13, 0.13, and 0.07 for TEB-O-Glc, TEB-OH, TEB-COO-Glc, and TEB-COOH. Urine samples were submitted to hydrolysis with β-glucuronidase, and the free compounds were quantified in the presence of deuterated TEB (TEB-d6) as the internal standard (IS), by multiple reaction monitoring (MRM) mode. The assay was linear in the ranges of 0.2-600 μg/L and 0.1-240 μg/L for TEB-OH and TEB-COOH, respectively; precision, accuracy, and the limit of quantification (LOQ) were <3.1%, 98-103%, and 0.3 μg/L for both analytes. An evaluation of matrix effects showed that the use of TEB-d6 controlled these sources of bias. The urinary levels of TEB-OH and TEB-COOH in specimens collected from farmers exposed to TEB ranged from 10 to 473 and from 3 to 159 μg/L, respectively. DOI: 10.1021/tx500291t PMID: 25255061 [Indexed for MEDLINE]

[16] J Expo Sci Environ Epidemiol. 2014 Nov;24(6):643-9. doi: 10.1038/jes.2014.14 Biological monitoring of exposure to tebuconazole in winegrowers. Fustinoni S(1), Mercadante R(1), Polledri E(1), Rubino FM(2), Mandic-Rajcevic S(2), Vianello G(2), Colosio C(2), Moretto A(3). Author information: (1)Department of Clinical Sciences and Community Health, University of Milano and Fondazione IRCCS Ca' Granda Ospedale Maggiore Policlinico, Milan, Italy. (2)Department of Health Sciences of the University of Milan, Laboratory for Analytical Toxicology and Metabolomics (LaTMA) and International Centre for Rural Health of the University Hospital San Paolo, Milan, Italy. (3)Department of Biomedical and Clinical Sciences, University of Milano, and International Centre for Pesticides and Health Risks Protection (ICPS), Luigi Sacco Hospital, Milano, Italy. Tebuconazole (TEB) is a fungicide widely used in vineyards and is a suspected teratogen for humans. The aim of this research was to identify urinary biomarkers and the best sampling time for the biological monitoring of exposure to TEB in agricultural workers. Seven vineyard workers of the Monferrato region, Piedemont, Italy, were investigated for a total of 12 workdays. They treated the vineyards with TEB for 1-2 consecutive days, one of them for 3 days. During each application coveralls, underwears, hand washing liquids and head coverings were used to estimate dermal exposure. For biomonitoring, spot samples of urine from each individual were collected starting from 24 h before the first application, continuing during the application, and again after the application for about 48 h. TEB and its metabolites TEB-OH and TEB-COOH were measured by liquid chromatography/triple quadrupole mass spectrometry. TEB contamination of coveralls and total dermal exposure showed median levels of 6180 and 1020 μg. Urinary TEB-OH was the most abundant metabolite; its excretion rate peaked within 24 h after product application (post 24 h). In this time frame, median levels of TEB-OH and TEB-COOH ranged from 8.0 to 387.8 μg/l and from 5.7 to 102.9 μg/l, respectively, with a ratio between the two metabolites of about 3.5. The total amount of urinary metabolites (U-TEBeq) post 24 h was significantly correlated with both TEB on coveralls and total dermal exposure (Pearson's r=0.756 and 0.577). The amount of metabolites excreted in urine represented about 17% of total dermal TEB exposure. Our results suggest that TEB-OH and TEB-COOH in post-exposure urine samples are promising candidates for biomonitoring TEB exposure in agricultural workers. DOI: 10.1038/jes.2014.14 PMID: 24619295 [Indexed for MEDLINE]

[17] Occup Environ Med. 2014 Feb;71(2):126-33. doi: 10.1136/oemed-2013-101490. Biomonitoring of organophosphate exposure of pesticide sprayers and comparison of exposure levels with other population groups in Thessaly (Greece). Koureas M(1), Tsakalof A, Tzatzarakis M, Vakonaki E, Tsatsakis A, Hadjichristodoulou C. Author information: (1)Department of Hygiene and Epidemiology, Faculty of Medicine, University of Thessaly, Larissa, Greece. OBJECTIVES: To evaluate the exposure of different population groups in Thessaly (Greece) to organophosphate pesticides (OPs) and investigate the dependence of exposure levels on pesticide application practices, personal protective and hygienic measures taken. METHODS: For the exposure assessment, four dialkyl phosphate (DAP) metabolites of organophosphate pesticides were quantified in spot urine samples of 77 pesticide sprayers, 75 residents of the studied agricultural area non-involved in agricultural activities and 112 urban residents who served as a control group. Structured questionnaires were used to record demographic characteristics, pesticide application parameters and protective measures taken. Univariate and multivariate analysis of the obtained cross-

sectional data was performed to identify potential risk factors associated with biomarker levels. RESULTS: It was found that total DAP median level in the sprayers' group was 24.9 µg/g creatinine (IQR: 13.0-42.1), while the rural and urban residents had significantly lower (p<0.001) levels of 11.3 µg/g creatinine (IQR: 5.3-18.7) and 11.9 µg/g creatinine (IQR: 6.3-20.3), respectively. In sprayers who had recently applied an OP pesticide (n=28), the median levels of DAP metabolites were 31.8 µg/g creatinine (IQR: 22.3-117.2). Logistic regression analysis showed that the use of full body coveralls while handling and spraying pesticides was significantly associated with lower DAP levels (OR 4.05, 95% CI 1.22 to 13.46). Also, changing clothes immediately after accidental contamination of clothing with pesticide amounts was found to be significantly associated with lower exposure levels (OR 4.04, CI 1.05 to 15.57). CONCLUSIONS: Our study findings confirm the increased exposure to OPs in pesticide sprayers and underline the importance of protective measures especially those that focus on dermal exposure mitigation. DOI: 10.1136/oemed-2013-101490 PMID: 24186943 [Indexed for MEDLINE]

[18] Sci Total Environ. 2014 Feb 1;470-471:282-9. doi: 10.1016/j.scitotenv.2013.09.021. Dermal & inhalation exposure of operators during fungicide application in vineyards. Evaluation of coverall performance. Tsakirakis AN(1), Kasiotis KM(1), Charistou AN(1), Arapaki N(1), Tsatsakis A(2), Tsakalof A(3), Machera K(4). Author information: (1)Laboratory of Pesticides Toxicology, Benaki Phytopathological Institute, 8 St. Delta Street, Kifissia 14561, Athens, Greece. (2)Laboratory of Toxicology, Department of Medicine, University of Crete, 71409 Heraklion, Greece. (3)Laboratory of Biochemistry, Medical School, University of Thessaly, Greece. (4)Laboratory of Pesticides Toxicology, Benaki Phytopathological Institute, 8 St. Delta Street, Kifissia 14561, Athens, Greece. Electronic address: k.machera@bpi.gr. In the present study the dermal and the inhalation exposure of five operators during fungicide applications in vineyards were determined. The produced exposure datasets can be used as surrogate for the estimation of the actual and the potential dermal as well as inhalation operator exposure levels for this application scenario. The dermal exposure was measured using the whole body dosimetry method while the inhalation exposure with the use of personal air sampling devices with XAD tubes located on the operator's breathing zone. Ten field trials were carried out by 5 different operators using a tractor assisted hand-held lance with spray gun at the Tanagra region of Viotia, Greece. An in-house GC-ECD analytical method was developed and validated for the determination of penconazole, which was the active substance (a.s.) of the fungicide formulation used in field trials. The mean recovery of field-fortified samples was 81%. The operator exposure results showed expected variability and were compared to those derived from the German model for prediction of operator exposure. The comparison of the 75th percentile values for an operator wearing personal protection equipment has shown that the measured levels were 2.2 times lower than those estimated by the German model. The levels of actual dermal exposure ranged from 2 to 19 mg/kg a.s. applied. The protection provided by the two types of coveralls was evaluated and in comparison to the existing reduction factors used for other types of PPE (coveralls) was found satisfactory for the operator under the conditions of the specific applications. DOI: 10.1016/j.scitotenv.2013.09.021 PMID: 24140699

[19] Environ Int. 2013 Oct;60:42-7. doi: 10.1016/j.envint.2013.07.016. Biomonitoring short- and long-term exposure to the herbicide terbuthylazine in agriculture workers and in the general population using urine and hair specimens. Mercadante R(1), Polledri E, Bertazzi PA, Fustinoni S. Author information: (1)Department of Clinical Sciences and Community Health, University of Milan and Fondazione IRCCS Ca' Granda Ospedale Maggiore Policlinico, Via S. Barnaba, 8-20122 Milan, Italy. The aim of this work was to evaluate short-term and long-term exposure to terbuthylazine (TBA) in agriculture workers (AW), rural residents (RR), and urban residents (UR) using urine and hair specimens. Twelve AW, 13 RR, and 17 UR were included in the study. Urine spot samples were collected with two different protocols. AW urine samples were collected before the application season (February, U0), at bedtime on the day of TBA application (March-May, U1), and prior to the next shift on the day after TBA application (U2). RR and UR urine samples were collected on any day during the application season (Ue). Hair samples were collected for all subjects before the application season (February, H0) and at the end of the season (June, H1). TBA and its metabolite desethylterbuthylazine (DET) were measured by liquid chromatography coupled with triple

quadrupole mass spectrometry detection. DET was exclusively found in urine, while TBA was mostly found in the hair. In the AW, the urinary levels of DET were not detected in the U0 samples, and they increased to median levels of 1.81 and 2.94μg/L in the U1 and U2 samples, respectively (p<0.001). In the RR and UR, DET was not detected in the Ue samples. In the UR, TBA was not detected in the H0 samples, and the median levels of TBA were 0.01ng/mg hair in both the AW and RR. In the H1 samples, the median TBA levels were not detected, 0.01, and 0.08ng/mg hair in the UR, RR, and AW, respectively (p<0.001). Urinary DET and hair TBA are promising candidates for biomonitoring short- and long-term exposure to TBA. The use of this herbicide in agriculture leads to exposure in rural residents. DOI: 10.1016/j.envint.2013.07.016 PMID: 23995105 [Indexed for MEDLINE]

[20] Int J Immunopathol Pharmacol. 2013 Apr-Jun;26(2):517-23. doi: 10.1177/039463201302600226. Evaluation by environmental monitoring of pesticide absorption in farm workers of 18 Italian tomato cultivations. Basilicata P(1), Simonelli A, Silvestre A, Lamberti M, Pedata P, Feola D, Acampora A, Pieri M, Sannolo N, Miraglia N. Author information: (1)Department of Experimental Medicine-Section of Hygiene, Occupational Medicine and Forensic Medicine, Second University of Naples, Naples, Italy. Tomato cultivation farms of Southern Italy were investigated in order to evaluate the general working conditions and the levels of exposure of farm workers to pesticides, during the mixing/loading and the application of pesticides on fields. Information on working modalities, personal protective equipment, etc. was collected using a questionnaire. Inhaling and cutaneous exposure levels were measured, and the estimated pesticide total absorbed dose was compared with Admissible Daily Intakes (ADIs). Field treatments were mainly carried out by using sprayers with open cab tractors, and, in 57.9 percent of cases, the pesticide mixture was manually prepared by mixing pesticides in a pail, often without using gloves (59.5 percent). The estimated pesticides absorbed doses varied in the range 0.56-2630.31 mg (mean value, 46.9 mg), and 20 percent of the measured absorbed doses exceeded ADIs. The findings obtained in the 18 examined farms show a worrying situation, suggesting the investigation of many more farms, so that a statistically significant picture of tomato cultivations in Southern Italy could be formed. Besides, the planning of training courses aimed to increase workers consciousness about health risks and how they can be prevented is advisable. DOI: 10.1177/039463201302600226 PMID: 23755768 [Indexed for MEDLINE]

[21] J Expo Sci Environ Epidemiol. 2012 Nov;22(6):593-600. doi: 10.1038/jes.2012.82. Levels and determinants of pesticide exposure in operators involved in treatment of vineyards: results of the PESTEXPO Study. Baldi I(1), Lebailly P, Rondeau V, Bouchart V, Blanc-Lapierre A, Bouvier G, Canal-Raffin M, Garrigou A. Author information: (1)University Bordeaux, ISPED, Laboratoire Santé Travail Environnement, Bordeaux, France. Isabelle.Baldi@isped.u-bordeaux2.fr Exposure assessment is a critical point for epidemiological studies on pesticide health effects. PESTEXPO study provides data on levels of exposure and their determinants in real conditions of pesticide use. We described levels of exposure in vineyards during treatment tasks (mixing, spraying and cleaning) and we analysed their determinants. Sixty-seven operators using dithiocarbamates or folpet were observed. Detailed information on the tasks (general conditions, operator, farm and equipment characteristics) were collected and dermal contamination was measured, using patches placed onto the skin on eleven body parts, and washing the hands at the end of each phase. The spraying phase represented roughly half of the contamination, whereas mixing and equipment cleaning accounted for 30% and 20% of the contamination, respectively. The main determinants of exposure were the number of phases, the characteristics of the equipment, the educational level of the operator and his status (farm -worker or -owner) and the general characteristics of the vines. Algorithms were built to estimate daily external contamination, according to these characteristics during mixing, spraying or equipment cleaning. With additional information of frequency and duration of use, they will enable to develop exposure indices usable in epidemiological studies on farmers' health. DOI: 10.1038/jes.2012.82 PMID: 22892809 [Indexed for MEDLINE]

[22] J Biol Regul Homeost Agents. 2012 Jul-Sep;26(3):439-45. Perception of occupational risk by rural workers in an area of central Italy. Antonucci A(1), Siciliano E, Ladiana D, Boscolo P, Di Sivo M. Author information: (1)Operative Unit of Occupational Medicine, University of Chieti-Pescara, Italy. andrea.antonucci@unich.it The aim of this study is to analyze the subjective perception of risks for rural workers in Abruzzo, an area of central Italy. A group of 273 workers were asked to fill in a questionnaire which included, apart from general information, questions relative to six different types of risks normally found in the field of agriculture. The types of risks considered were: falling from a height, manually moving loads, overturning/accident whilst driving an agricultural tractor, noise and vibration, use of pesticides, the risk of being cut/injured. The workers were requested to assess, on a scale of 1 to 3, both the probability of an accident taking place and the consequent damage which could result from each of the risks considered. The assessment of the risks provided by the workers was related to the objective assessment of the risks carried out by the study group, also on the basis of objective data provided by INAIL (Italian insurance company) indexes, to highlight the eventual under/over estimations of risk. Furthermore, the possible correlation was evaluated between having received specific training regarding work safety and the workers perception of the risk. The results showed that approximately 11 percent of the workers do not consider their job as being dangerous; the risk perceived by the workers is higher for accidents that cause an immediate injury compared to those which cause professional illnesses, except the risk deriving from noise/vibrations. A direct correlation was found between the job as being dangerous and having attended courses on accident prevention. PMID: 23034263 [Indexed for MEDLINE]

[23] Toxicol Lett. 2012 Apr 25;210(2):189-97. doi: 10.1016/j.toxlet.2012.01.017. Farmers' exposure to herbicides in North Italy: assessment under real-life conditions in small-size rice and corn farms. Rubino FM(1), Mandic-Rajcevic S, Ariano E, Alegakis A, Bogni M, Brambilla G, De Paschale G, Firmi A, Minoia C, Micoli G, Savi S, Sottani C, Somaruga C, Turci R, Vellere F, Tsatsakis A, Colosio C. Author information: (1)LaTMA Laboratory for Analytical Toxicology and Metabolomics, Department of Medicine, Surgery and Dental Sciences, University of Milan, San Paolo University Hospital, Via di Rudinì 8 20124 Milan, Italy. Although rice and corn are two main cash crops in Lombardy (North Italy) and their cultivation involves several thousands of farmers, risk assessment of pesticide exposure is rarely done, especially in small and medium sized enterprises. With the use of pads for environmental monitoring (OECD protocol), of pre- and post-exposure 24h urine collection for biological monitoring and of hand wash for hands' exposure, we measured the exposure of 28 agricultural workers to propanil and terbuthylazine in real-life working conditions. In propanil applicators, median daily exposure on the clothes was 73.5μmol per worker, while the exposure on the skin was 22.4μmol. For terbuthylazine, these exposures were 37.2μmol and 0.86μmol per worker, respectively. Median excretion of the propanil metabolite (3,4-dichloroaniline) after exposure was 84nmol in 24h urine, and 13nmol for the metabolite of terbuthylazine. Risk assessment performed by comparing to the AOELs of the applied active ingredients with an estimated internal dose, obtained based on the measured levels of skin and hand exposure and the percentage of dermal absorption of the active ingredients considered showed that 4 propanil workers, and no terbuthylazine workers, were overexposed. Our study helps define and confirm relationships between different exposure determinants, which can be used in the development of tools for risk assessment of exposure to pesticides in small and medium sized enterprises. DOI: 10.1016/j.toxlet.2012.01.017 PMID: 22306369 [Indexed for MEDLINE]

[24] Ann Occup Hyg. 2010 Jun;54(4):443-52. doi: 10.1093/annhyg/meq014. Development of a Task-Exposure Matrix (TEM) for Pesticide Use (TEMPEST). Dick FD(1), Semple SE, van Tongeren M, Miller BG, Ritchie P, Sherriff D, Cherrie JW. Author information: (1)Environmental and Occupational Medicine, Population Health Section, Division of Applied Health Sciences, School of Medicine and Dentistry, University of Aberdeen, Foresterhill, Aberdeen, AB25 2ZD, UK. f.dick@abdn.ac.uk INTRODUCTION: Pesticides have been associated with increased risks for a range of conditions including Parkinson's disease, but identifying the agents responsible has proven challenging. Improved pesticide exposure estimates would increase the power of epidemiological studies to detect such an association if one exists. METHODS: Categories of pesticide use were identified from the tasks reported in a previous community-based case-control study in Scotland. Typical pesticides used in

each task in each decade were identified from published scientific and grey literature and from expert interviews, with the number of potential agents collapsed into 10 groups of pesticides. A pesticide usage database was then created, using the task list and the typical pesticide groups employed in those tasks across seven decades spanning the period 1945-2005. Information about the method of application and concentration of pesticides used in these tasks was then incorporated into the database. RESULTS: A list was generated of 81 tasks involving pesticide exposure in Scotland covering seven decades producing a total of 846 task per pesticide per decade combinations. A Task-Exposure Matrix for PESTicides (TEMPEST) was produced by two occupational hygienists who quantified the likely probability and intensity of inhalation and dermal exposures for each pesticide group for a given use during each decade. CONCLUSIONS: TEMPEST provides a basis for assessing exposures to specific pesticide groups in Scotland covering the period 1945-2005. The methods used to develop TEMPEST could be used in a retrospective assessment of occupational exposure to pesticides for Scottish epidemiological studies or adapted for use in other countries.

[25] Ann Occup Hyg. 2009 Aug;53(6):573-84. doi: 10.1093/annhyg/mep032. Epub 2009 May 27. Dermal exposure of pesticide applicators as a measure of coverall performance under field conditions. Machera K(1), Tsakirakis A, Charistou A, Anastasiadou P, Glass CR. Author information: (1)Laboratory of Pesticides Toxicology, Department of Pesticides Control and Phytopharmacy, Benaki Phytopathological Institute, 8 St Delta Street, GR-145 61 Kifissia, Athens, Greece. machera@otenet.gr In this study, the field performance of two coverall designs used by pesticide applicators was determined. Two coverall types were selected based on data from previously conducted comfort testing under field conditions in southern Europe. Dermal exposure was measured during 22 applications conducted with 11 operators using similar hand-held spray guns in greenhouse pepper crops in the Ierapetra region of Crete, Greece. One of the coverall designs studied was made from a cotton/polyester material treated with a water-repellent Resist Spills(R) finish, which was compared in the field study to a coverall of similar design, but using a woven, untreated cotton material. An in-house analytical method was developed and validated for determining residues of the active substance (a.s.) malathion on the dosimeters. The derived levels of dermal exposure were used as a measure of the protection provided by the two types of coveralls. In addition, by comparing the total amount of the a.s. recovered from outer and inner dosimeters (potential dermal exposure = 238.8 mg kg(-1) a.s. for the cotton coverall and 160.44 mg kg(-1) a.s. for the Resist Spills coverall), a value could be determined for the degree of coverall penetration. The mean penetration (milligrams per kilogram a.s.) of the outer coveralls, calculated as a percentage of the total contamination, was 0.4% for the water-repellent coverall and 2.3% for the cotton coverall. The mean recovery from the laboratory and field-fortified samples was >91 and 74%, respectively and used as the main criterion for quality control of the analytical data. Under the field trial conditions evaluated, both the coverall designs gave better protection than the default values used in the most relevant predictive exposure model. Therefore, they could be considered as appropriate tools of personal protection when both comfort and field performance is taken into account under the specific application scenario. DOI: 10.1093/annhyg/mep032 PMID: 19474075 [

[26] Ann Occup Hyg. 2009 Jan;53(1):69-81. doi: 10.1093/annhyg/men072 Exposure to pesticides in open-field farming in France. Lebailly P(1), Bouchart V, Baldi I, Lecluse Y, Heutte N, Gislard A, Malas JP. Author information: (1)Groupe Régional d'Etudes sur le CANcer (EA-1772), IFR146 ICORE, Université de Caen Basse-Normandie, 14076 Caen Cedex 5, France. p.lebailly@baclesse.fr OBJECTIVES: Identification of parameters associated with measured pesticide exposure of farmers in open-field farming in France. METHODS: Open-field volunteer farmers were monitored during 1 day use of the herbicide isoproturon on wheat and/or barley during the winters 2001 (n = 9) or 2002 (n = 38) under usual conditions of work. The whole-body method was used to assess potential dermal exposure using coveralls and cotton gloves. Mixing-loading and application tasks were assessed separately with 12 different body areas (hands, arms, forearms, legs, chest, back and thighs) measured for each task (mixing-loading and application separately). RESULTS: Daily potential dermal exposure to isoproturon ranged from 2.0 to 567.8 mg (median = 57.8 mg) in 47 farmers. Exposure during mixing-loading tasks accounted for 13.9-98.1% of the total exposure (median = 74.8%). For mixing-loading, hands and forearms were the most contaminated body

areas accounting for an average of 64 and 14%, respectively. For application, hands were also the most contaminated part of the body, accounting for an average of 57%, and thighs, forearms and chest or back were in the same range as one another, 3-10%. No correlations were observed between potential dermal exposure and area sprayed, duration of spraying or size of the farm. However, a significant relationship was observed between exposure and the type of spraying equipment, with a rear-mounted sprayer leading to a higher exposure level than trailer sprayers. Technical problems, particularly the unplugging of nozzles, and the numbers mixing-loading or application tasks performed were also significantly related with higher levels of exposure. CONCLUSIONS: The main results obtained in this study on a large number of observation days are as follows: (i) the mixing-loading step was the most contaminated task in open field accounting for two-thirds of the total daily exposure, (ii) no positive correlation was noted with classically used pesticide-related parameters: farm area, area sprayed and duration of application and (iii) relevant parameters were the type of spraying equipment, the type and number of tasks and technical problems or cases of overflowing. DOI: 10.1093/annhyg/men072 PMID: 19022871

[27] Biological monitoring and questionnaire for assessing exposure to ethylenebisdithiocarbamates in a multicenter European field study. Fustinoni S(1), Campo L, Liesivuori J, Pennanen S, Vergieva T, van Amelsvoort L, Bosetti C, Van Loveren H, Colosio C. Author information: (1)Department of Occupational and Environmental Health, University of Milan and Fondazione IRCCS Ospedale Maggiore Policlinico, Mangiagalli e Regina Elena, Milan, Italy. silvia.fustinoni@unimi.it This study deals with pesticide exposure profile in some European countries with a specific focus on ethylenebisdithiocarbamates (EBDC). In all, 55 Bulgarian greenhouse workers, 51 Finnish potato farmers, 48 Italian vineyard workers, 42 Dutch floriculture farmers, and 52 Bulgarian zineb producers entered the study. Each group was matched with a group of not occupationally exposed subjects. Exposure data were gained through self-administered questionnaires and measuring ethylenethiourea (ETU) in two spot urine samples collected, respectively, before the beginning of seasonal exposure (T0), and after 30 days, at the end of the exposure period (T30). Controls underwent a similar protocol. Study agriculture workers were involved in mixing and loading pesticides, application of pesticide mixture with mechanical or manual equipments, re-entry activities, and cleaning equipments. Chemical workers were involved in synthesis, quality controls, and packing activities. The number of pesticides to whom these subjects were exposed varied from one (zineb production) to eight (potato farmers). The use of personal protective devices was variegate and regarded both aerial and dermal penetration routes. EBDC exposure, assessed by T30 urinary ETU, was found to follow the order: greenhouse workers, zineb producers, vineyard workers, potato farmers, floriculture farmers with median levels of 49.6, 23.0, 11.8, 7.5, and 0.9 microg/g creatinine; the last group having ETU at the same level of controls (approximately 0.5 microg/g creatinine). Among agriculture workers, pesticide application, especially using manual equipment, seems to be the major determinant in explaining internal dose. Although the analysis of self-administered questionnaires evidenced difficulties especially related to lack and/or poor quality of reported data, biological monitoring confirms to be a powerful tool in assessing pesticide exposure. DOI: 10.1177/0960327108100003 PMID: 19042950

[28] Measuring determinants of occupational health related behavior in Flemish farmers: an application of the Theory of Planned Behavior. Colémont A(1), Van den Broucke S. Author information: (1)Catholic University Leuven. ariane.colemont@psy.kuleuven.be PROBLEM: Preventive interventions to reduce occupational injuries and health problems in farmers require the identification of factors that contribute to unsafe and health damaging behavior. This paper describes the development and validation of a self-report questionnaire, which measures the determinants of occupational health-related behaviors in farmers. METHOD: A representative sample of 283 Flemish farmers completed a provisional 135 item questionnaire based on the Theory of Planned Behavior, measuring four behaviors related to occupational health (machinery use, animal handling, preventing falls, and pesticide use), as well as the intentions, attitudes, perceived social norms, and self-efficacy for each of these behaviors. RESULTS: The fit indices of the Confirmatory Factor Analysis turned out not to be sufficient to reproduce the dimensions of the TPB. Therefore exploratory factor analysis was use to determine the underlying dimensions. Principal Component Analyses (PCA) on the behavioral items yielded single component solutions explaining a

considerable proportion of the variance for each behavior and for behavioral intentions. Principal component analyses toward an a priori three-component structure reflecting the TPB dimensions did not produce sufficient congruence for the determinants of the four behaviors. Subsequent Varimax rotations and discarding of redundant items resulted in three component solutions explaining 50% to 69% of the variance in the determinants of each behavior, corresponding with the dimensions of the TPB. Internal consistencies ranged from .25 to .89. Scale scores accounted for a significant proportion of the variance in intention and self-reported behavior. IMPACT: The study demonstrates the validity of the TPB in predicting behavior related to occupational safety and health in farmers, and provides a valid and reliable questionnaire to measure the cognitive concepts featured in this model. IMPACT ON INDUSTRY: Both authors share the same view on this study's impact on industry. In recent years, efforts have been made to create awareness among farmers about occupational hazards, and to encourage farmers to perform safer and healthier behavior. However, only a limited number of these interventions have proven to be successful. A possible reason for this relative lack of success is that interventions typically focus on risk analysis and raising awareness, whereas the literature on preventive health behavior change indicates that knowledge and awareness with regard to possible health risks are neither necessary nor sufficient to change behavior. To raise the effectiveness of prevention programs, other relevant determinants of behavior need to be addressed as well, such as: attitudes, perceived social norms, self efficacy, and elements of the physical environment that elicit or reinforce behavior. These determinants play a key role in psychological models of health related behavior, such as the Theory of Planned Behavior. Thus far, the use of these models within agricultural settings is fairly limited. This study demonstrates the validity of the Theory of Planned Behavior in predicting behavior related to occupational safety and health in farmers, and provides a valid and reliable questionnaire to measure the cognitive concepts featured in this model. DOI: 10.1016/j.jsr.2007.12.001 PMID: 18325417

[29] Changes in serum markers indicative of health effects in vineyard workers following exposure to the fungicide mancozeb: an Italian study. Colosio C(1), Fustinoni S, Corsini E, Bosetti C, Birindelli S, Boers D, Campo L, La Vecchia C, Liesivuori J, Pennanen S, Vergieva T, Van Amelsvoort LG, Steerenberg P, Swaen GM, Zaikov C, Van Loveren H. Author information: (1)Department of Occupational and Environmental Health of the University of Milan, S. Paolo Hospital Unit, Milan, Italy. claudio.colosio@unimi.it The aim of this study was to investigate the health effects induced by exposure to the fungicide mancozeb in Italian vineyard workers. Ninety-three Italian subjects entered the study - 48 vine-growers intermittently exposed to mancozeb and 45 healthy controls. The subjects were investigated three times: before the seasonal application of pesticides (T0), 30 days after the beginning of the application period (T30), and 45 days after T0 (T45). At T0 the comparison between agricultural workers and controls showed a higher prevalence of cold or flu symptoms, a statistically significant lower percentage of monocytes, higher absolute count of T lymphocytes, CD4 and natural killer cells, and lower plasma levels of IgA and IgM in workers. Such differences were not confirmed at T30 and T45. In fact at T30 in exposed workers, besides a significant increase of urinary ethylenethiourea, confirming mancozeb exposure, T lymphocytes, CD4 and natural killer cells, IgA and IgM returned to values comparable to those observed in controls. Moreover, no other differences in clinical signs, haematological, and immune parameters, such as the immune functional capability evaluated as a response to hepatitis B vaccination, was observed. Altogether the differences between exposed and controls were not consistently correlated to any clinical impairment and suggest that the seasonal application of mancozeb does not pose a significant health risk to exposed subjects. DOI: 10.1080/13547500701441315 PMID: 17852083

[30] Commun Agric Appl Biol Sci. 2007;72(2):87-93 Personal protective equipment for registration purposes of pesticides. Gerritsen-Ebben MG(1), Brouwer DH, van Hemmen JJ. Author information: (1)TNO Quality of Life, Food and Chemical Risk Analysis, Chemical Exposure Assessment, Zeist, The Netherlands. Regulatory authorities in North America, Europe and Australia use different approaches for the estimation of exposure reduction effectiveness of personal protective equipment (PPE) in registration processes of agrochemical pesticides. TNO has investigated current views and facts for the use of default values and set up a discussion paper which can be used as a starting point to achieve an internationally harmonised set of PPE

protection factors for regulatory use. For inhalation exposure Loading it is proposed to use the assigned protection factors (APF) as deduced by BSI (British Standard Institution) and ANSI (American National Standards Institution). Since these values are somewhat variance and since in agricultural settings efficient control and proper training and education with respect to inhalation protection devices is generally absent, it is good to err on the safe side and to use the Lowest of both values, if available. For dermal exposure Loading differentiations are made for operators and re-entry workers and further for hand and body protection. Next to this the restrictions and framework for the use of the proposed defaults are very relevant. Oral exposure loading is only considered in special cases where dermal exposure may be relatively high and the hand-mouth shunt may lead to appreciable oral exposure loading. The presented defaults for PPE have been discussed with experts of regulatory authorities and industry, but a formal discussion still has to take place. This needs to be done on EU level between Member States. The current proposal is based on state-of-the-art knowledge and policy considerations, but further research is needed to better underpin the proposed values and/or to adapt them. PMID: 18399428

[31] Neuropsychiatric symptoms in past users of sheep dip and other pesticides. Solomon C(1), Poole J, Palmer KT, Peveler R, Coggon D. Author information: (1)MRC Epidemiology Resource Centre, Southampton General Hospital, Southampton, UK. OBJECTIVES: To explore the prevalence and pattern of neuropsychiatric symptoms in past users of sheep dip and other pesticides. METHODS: From a postal survey of men born between 1933 and 1977 and resident in three rural areas of England and Wales (response rate 31%), data were obtained on lifetime history of work with pesticides, neurological symptoms in the past month, current mental health and tendency to be troubled by non-neurological somatic symptoms (summarised as a somatising tendency score). Risk factors for current neuropsychiatric symptoms were assessed by modified Cox regression. RESULTS: Data were available for 9844 men, including 1913 who had worked with sheep dip, 832 with other insecticides but not sheep dip and 990 with other pesticides but never with sheep dip or insecticides. Neurological symptoms were consistently 20-60% more common in past users of sheep dip than in men who had never worked with pesticides, but their prevalence was also higher in men who had worked only with pesticides other than sheep dip or insecticides. They clustered strongly within individuals, but this clustering was not specific to men who had worked with sheep dip. Reporting of three or more neurological symptoms was associated with somatising tendency (prevalence ratio (PR) 15.0, 95% CI 11.4 to 19.5, for the highest vs the lowest category of somatisation) and was more common in users of sheep dip (PR 1.3, 95% CI 1.0 to 1.6), other insecticides (PR 1.4, 95% CI 1.0 to 1.8) and other pesticides (PR 1.3, 95% CI 1.0 to 1.7) than in non-users. Among users of sheep dip, prevalence was higher in men who had dipped most often, but not in those who had worked with sheep dip concentrate. Past use of pesticides was not associated with current anxiety or depression. CONCLUSION: Neurological symptoms are more common in men who have worked with sheep dip, but the association is not specific to sheep dip or insecticides. A toxic cause for the excess cannot be ruled out, but several features of our observations suggest that psychological mechanisms have a role. DOI: 10.1136/oem.2005.023879 PMCID: PMC2078460 PMID: 17095551

[32] J Expo Sci Environ Epidemiol. 2006 Mar;16(2):115-24. Pesticide contamination of workers in vineyards in France. Baldi I(1), Lebailly P, Jean S, Rougetet L, Dulaurent S, Marquet P. Author information: (1)Laboratoire Santé Travail Environnement, Institut de Santé Publique d'Epidémiologie et de Développement, IFR 99, Université Victor Segalen Bordeaux, France. Isabelle.Baldi@isped.u-bordeaux2.fr In order to build tools to quantify exposure to pesticides of farmers included into epidemiological studies, we performed a field study in Bordeaux vineyards during the 2001 and 2002 treatment seasons to identify parameters related to external contamination of workers. In total, 37 treatment days were observed in tractor operators corresponding to 65 mixing operations, 71 spraying operations and 26 equipment cleaning. In all, four operators with backpack sprayers and seven re-entry workers were also monitored. We performed both detailed observations of treatment characteristics on the whole day and pesticide measurements of external contamination (dermal and inhalation) for each operation. The median dermal contamination was 40.5 mg of active ingredient per day for tractor operators, 68.8 mg for backpack sprayers and 1.3 mg for vineyard workers. Most of the contamination was observed on the hands (49% and 56.2% for mixing and spraying,

respectively). The median contribution of respiratory route in the total contamination was 1.1%. A cleaning operation resulted in a 4.20 mg dermal contamination intermediate between a mixing (2.85 mg) and a spraying operation (6.13 mg). Farm owners experienced higher levels than workers and lower contaminations were observed in larger farms. The contamination increased with the number of spraying phases and when equipment cleaning was performed. Types of equipment influenced significantly the daily contamination, whereas personal protective equipment only resulted in a limited decrease of contamination. DOI: 10.1038/sj.jea.7500443 PMID: 16175199

[33] Regul Toxicol Pharmacol. 2005 Nov;43(2):141-9 Applicator exposure to acetochlor based on biomonitoring. Gustin CA(1), Moran SJ, Fuhrman JD, Kurtzweil ML, Kronenberg JM, Gustafson DI, Marshall MA. Author information: (1)Monsanto Company, St. Louis, MO, USA. christophe.gustin@monsanto.com Biomonitoring was used to assess the combined dermal, oral, and inhalation exposure associated with the agricultural use of Harness Plus, an emulsifiable concentrate formulation of the herbicide acetochlor. Twenty Spanish farmers handled and applied acetochlor to maize in the spring of 2003, following the product label recommendations. Open- and closed-cabin applications were equally represented. Urine was collected during six consecutive days, starting the day prior to application. Daily composites were analyzed for 2-ethyl-6-methyl-aniline, a common chemophore representing the major urinary acetochlor metabolites. All applicators showed detectable concentrations in urine after application. Although, the open-cabin applicators treated fewer hectares, they showed significantly higher exposure compared to the closed-cabin applicators (average exposure: 0.004 and 0.002 mg/kg bw/day, respectively). Linear regression analysis suggested that untracked incidents had a significant impact on the total exposure. Other events that may have contributed to the observed exposure are repair of faulty equipment, accidental spillages, splashes, and inadequate use of protective gloves. The average margins of exposure (MOE) for farmers ranged from 23,000 (open cabin) to about 44,000 (closed cabin). For professional applicators the MOEs were 10-fold lower. These MOEs clearly indicate that no adverse health effects should be expected from agricultural acetochlor applications. DOI: 10.1016/j.yrtph.2005.06.015 PMID: 16169643 [Indexed for MEDLINE]

[34] Ann Occup Hyg. 2005 Jun;49(4):345-50 Pesticide residues on the external surfaces of field crop sprayers: occupational exposure. Ramwell CT(1), Johnson PD, Boxall AB, Rimmer DA. Author information: (1)Cranfield University, Shardlow Hall, Shardlow, Derby DE72 2GN, UK. c.ramwell@csl.gov.uk There is a general perception, amongst farmers and researchers, that post-application residues on the external surfaces of sprayers are negligible compared with residues remaining on the internal surfaces, although there have been few attempts to verify this assumption. The aim of this study was to investigate, using cotton glove sampling, operator exposure resulting from three typical operations: entering and working in the cab, general handling of the sprayer and maintenance practices. Samples were analysed for azoxystrobin, carbendazim, chlorothalonil, cyanazine, cypermethrin, epoxiconazole, flusilazole, isoproturon, kresoxim-methyl, metazachlor, pendimethalin, pirimicarb and tebuconazole. Isoproturon, pendimethalin, chlorothalonil and carbendazim were detected >1 mg per glove pair on the cotton gloves, but on the whole there was variability in residue levels between and within compounds. Comparison of results with occupational exposure limits indicated that residues were such that they may not necessarily be considered insignificant. It may therefore be prudent to develop more efficient methods for the external decontamination of sprayers and/or raise awareness of the findings. DOI: 10.1093/annhyg/meh101 PMID: 15650013

[35] Arch Environ Contam Toxicol. 2005 Jan;48(1):127-34 Evaluation of skin and respiratory doses and urinary excretion of alkylphosphates in workers exposed to dimethoate during treatment of olive trees. Aprea C(1), Terenzoni B, De Angelis V, Sciarra G, Lunghini L, Borzacchi G, Vasconi D, Fani D, Quercia A, Salvan A, Settimi L. Author information: (1)Laboratorio di Sanità Pubblica, Azienda USL 7, Strada del Ruffolo, Siena, Italy. maaprea@tin.it This article describes a study of exposure to dimethoate during spraying of olive trees in Viterbo province in central Italy. Airborne concentrations of dimethoate were in the range 1.5 to 56.7 nmol/m(3). Total skin contamination was in the range 228.4 to 3200.7 nmol/d and averaged 96.0% +/-

3.6% of the total potential dose. Cotton garments afforded less skin protection than waterproof ones, which were in turn associated with higher skin contamination than disposable Tyvek overalls. Total potential doses and estimated absorbed doses, including their maxima, were below the acceptable daily intake of dimethoate, which is 43.6 nmol/kg body weight (b.w.). Urinary excretion of alkylphosphates was significantly higher than in the general population, increasing with exposure and usually showing a peak in the urine sample collected after treatment. Metabolite concentrations were influenced by the type of individual protection used: minimum levels were associated with the closed cabin and maximum levels with absence of any respiratory or hand protection. Urinary alkylphosphates showed a good correlation with estimated absorbed doses and are confirmed as sensitive biologic indicators of exposure to phosphoric esters. DOI: 10.1007/s00244-004-0073-5 PMID: 15657814

[36] [Risk profiles in agriculture: activities in the region of Lombardy]. [Article in Italian] Tiramani M(1), Ariano E, Birindelli S, Ronchin M, Savi S, Carreri V, Colosio C, Maroni M. Author information: (1)International Centre for Pesticides and Health Risk Prevention (ICPS), Ospedale Luigi Sacco, MI. Agriculture represents a very complex scenario that needs proper tools. To this aim, in the frame of Special Project "La Prevenzione nell'impiego di Antiparassitari in Agricoltura" promoted by the Region of Lombardy, profiles of exposure and/or risk have been identified as valid approach able to define particular conditions of exposure and risk for the operator in particular settings. The evaluation necessarily needs to identify the most important parameters affecting exposure and their extent on magnitude of exposure. Therefore, field studies should be further performed in order to confirm and improve the profile. The identification of profiles of exposure and risk is an activity still in progress that need to be carefully set up and standardised. The team involved in the study identified priorities deserving much attention in Lombardy, and planned a three years programme aimed at define the profile of exposure and risk in viticulture, maize crop, rice growing, nursery gardening, horticulture in greenhouses, maintenance of gardens, and poplar growing. PMID: 14979179

[37] [Pesticide application practices in agricultural workers]. [Article in Spanish] García AM(1), Ramírez A, Lacasaña M. Author information: (1)Departamento de Medicina Preventiva y Salud Pública, Bromatología, Toxicología y Medicina Legal, Universitat de València. anagar@uv.es OBJECTIVE: We describe the sociodemographic characteristics and determinants of pesticide exposure in agricultural workers applying pesticides. METHODS: The workers selected were included in a case-control study carried out in the Autonomous Community of Valencia in Spain. Contact was made by telephone and individuals who had been involved in agricultural work during the relevant period of exposure were interviewed face-to-face to gain information on the following determinants of pesticide exposure: crops and periods worked, mixing of products, treatment equipment, participation in the washing of equipment, use of personal protection during the treatments and knowledge of the risks of pesticide exposure. RESULTS: Eighty-nine workers, aged between 16 and 46 years old, were included. Most of the interviewees had primary education or less. Pesticide treatments were mostly applied on high crops (82%), with manual equipment (61%) and throughout the year (45%). Workers frequently performed tasks involving additional exposure to pesticides (mixing chemicals, 66%, or washing equipment, 60%). Sixty-five percent of the workers used no personal protection or used it defectively. No differences were found in personal protection use according to age, family income or education. Most of the workers (90%) reported knowledge of the health risks of pesticide exposure and 21% of them rated the risk as null. CONCLUSIONS: Workers involved in pesticide application use personal protection measures very defectively. There is a clear need to develop specific prevention programs for these workers. The determinants of pesticide exposure in agricultural workers described in this study should be properly assessed in epidemiological studies of the health effects of pesticides on agricultural workers. DOI: 10.1016/s0213-9111(02)71667-1 PMID: 12057179

[38] Personal air sampling and biological monitoring of occupational exposure to the soil fumigant cis-1,3-dichloropropene. Brouwer EJ(1), Verplanke AJ, Boogaard PJ, Bloemen LJ, Van Sittert NJ, Christian FE, Stokkentreeff M, Dijksterhuis A, Mulder A, De Wolff FA. Author information: (1)Coronel Laboratory for Occupational and Environmental Health, Department of

Human Toxicology, Academic Medical Center, University of Amsterdam, PO Box 22700, 1100 DE Amsterdam, The Netherlands. OBJECTIVES: To assess exposure of commercial application workers to the nematocide cis-1,3-dichloropropene (cis-DCP). METHODS: The study was conducted during the annual application season, August to 15 November, in the starch potato growing region in The Netherlands. 14 Application workers collected end of shift urine samples on each fumigation day (n=119). The mercapturic acid metabolite N-acetyl-S-(cis-3-chloro-2-propenyl)-L-cysteine (cis-DCP-MA) in urine was used for biological monitoring of the cis-DCP uptake. Inhalatory exposure was assessed by personal air sampling during a representative sample (n=37) of the fumigation days. Extensive information was collected on factors of possible relevance to the exposure and the application workers were observed for compliance with the statutory directions for use. The inhalatory exposure during all fumigation days was estimated from the relation between the personal air sampling data and the biological monitoring data. Exposure levels were correlated with the general work practice. The fumigation equipment and procedures were in accordance with the statutory directions of use, with the exception of the antidrip systems. Two antidrip systems were used: antidrip nozzles or a compressed air system. RESULTS: The geometric mean exposure of the application workers was 2.7 mg/m(3) (8 hour time weighted average); range 0.1-9.5 mg/m(3). On 25 days (21%) the exposure exceeded the Dutch occupational exposure limit (OEL) of 5 mg/m(3). This could mainly be explained by prolonged working days of more than 8 hours. The general work practice of the application workers was rated by the observers as good or poor. No difference in exposure to cis-DCP was found in the use of none, one, or two antidrip systems. Malfunctioning of the antidrip systems and lack of experience with the compressed air system were identified as possible causes for the lack of effectiveness of these antidrip systems. The use of personal protection was not always in accordance with the statutory directions of use. Dermal exposure to liquid cis-DCP was found four times during repair and maintenance, but the biological monitoring data did not suggest a significant increase in cis-DCP uptake. CONCLUSIONS: The application of cis-DCP in the potato growing industry can be performed at exposure concentrations below the Dutch OEL of 5 mg/m(3) if the working days are limited to 8 hours. An injector equipped with either kind of antidrip system which is in good working order, as well as the consistent use of personal protection in accordance with the statutory directions of use, may ensure exposure concentrations below the Dutch OEL. DOI: 10.1136/oem.57.11.738 PMCID: PMC1739889 PMID: 11024197 [Indexed for MEDLINE]

Federico Maria Rubino, Daniele Puri, Mario Fargnoli, Mara Lombardi, Stefan Mandic-Rajcevic and Claudio Colosio. **Sicurpest: a prototype of a user-friendly tool for preventive risk assessment of pesticide use in agriculture. Toxics (2025) doi:**

Supplementary material 2

Model construction of farmers' exposure to pesticides and algorithm's construction

Figure S2.1 shows the block scheme of the exposure and risk assessment calculation.

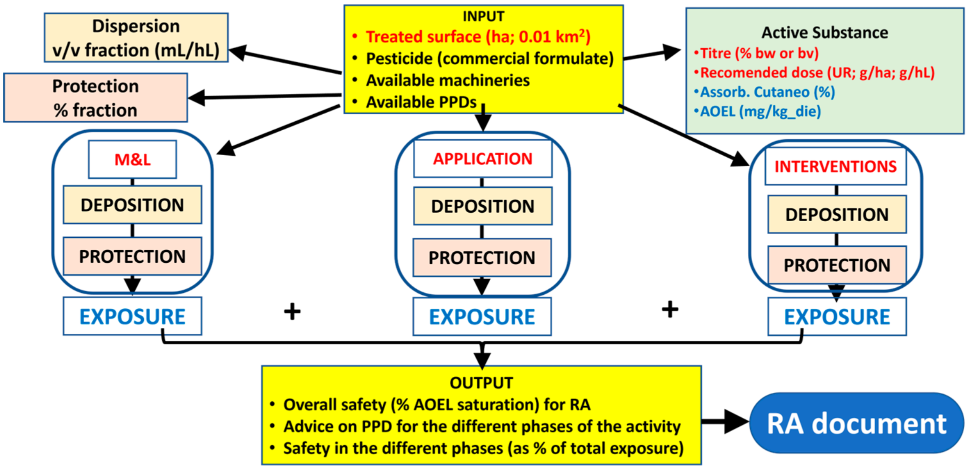

Figure S2.1. Block scheme of the exposure and risk assessment calculation with the Sicurpest tool.

Pesticide use

The amount of pesticide used to treat a specific area of the agricultural estate depends on:

- The actual size of the treated area (in the EU, in hectares, one hectare being 10,000 m2, and approx. 4 Imperial acres being one hectare);
- The Use Rate of the employed product, expressed in grams per hectare (g/ha). The Use Rate is the key efficacy parameter of an active substance. However, the same active substance can be contained in different formulations and products at different concentrations (usually expressed in g/100mL) or as percent mass ratio (% by weight).

The amount of actual pesticide (Active Ingredient, A.I.) is calculated as:

Employed Amount = surface of treated area (ha) * UR (g/ha) (**1**)

(g) (ha) * (g/ha)

The employed Amount refers to the weight formulated product. UR information is reported as lists or tables for each cultivated crop and fought pest, in the approved technical document that is mandatorily attached to the box or bottle and printed on it.

What is useful for Risk Assessment is the actual amount of A.I. in the manipulated product

Employed Amount (A.I.) = Employed Amount * A.I. conc. in product (**2**)

(g) (g) * (% or g/100 mL)

There is little homogeneity in the units employed to report the UR of the formulated product, since it depends on the physical nature of the formulation. Concentrated liquid solutions or dispersions are expressed (either or both) as grams or as millilitres per hectare. This allows measuring the concentrated product by volume or by weight. Powders and other solid forms are expressed as grams or kilograms. Some recent formulates (such as soluble bags) are sold in fixed amount to treat a defined area (usually one hectare, at the lowest UR) and multiple bags can be added to prepare the necessary amount.

The volume of water where the product is diluted or suspended is usually 1 hectolitre (1 hL = 100 L) per hectare. This dilution can vary from 0.5 hL/ha to 2 hL/ha.

Pesticide deposition upon spraying

The amount of sprayed pesticide used to treat a specific area of the agricultural estate that does not reach the crops, but deposits on the operator depends on:

- The volume fraction of water solution that reaches the operator, or Deposition Rate (as parts-per-million; 1 ppm = 0.1 mL / hL) and
- The concentration of pesticide in the sprayed solution (see above)

The Deposition Rate depends on the modality of pesticide application, which, in turn, depends on the type of crop and fought pest, on the landscape and on the employed equipment.

In particular, weed-killing entails dripping or spraying the solution vertically towards the ground from a very short distance. In this case, the Deposition Rate is itself very low and it is unlikely that the drops of solution do not fall from the machinery to the ground and drift horizontally off-target. On the contrary, insecticides and fungicides on bush and tree crops are applied by spraying at distance and entails the possibility that higher volume fractions of the solution drift off-target and can deposit on the operators.

Actual deposition on the operators depends on the presence of shielding between the source (the off-target spray) and the operator's body. Different modes of pesticide application entail different efficacy of shielding. Spraying from backpack sprayers (volume between 10 and 20 L), manually towing pipes on the ground and from open-cockpit tractors entail very high deposition for essential lack of shielding. Spraying from closed-cockpit tractors entails higher shielding, particularly when cockpit ventilation is performed by air filtration on active charcoal filters.

Deposited Amount is calculated as the product of Applied Amount and Deposition Efficiency of the application modality.

$$\underset{\text{(mg)}}{\text{Deposited Amount}} = \underset{\text{(g or kg) * (ppm, or mg/kg)}}{\text{Applied Amount * Deposition Efficiency}} \quad (3)$$

Worker shielding with the use of personal protective devices.

The next tier of worker protection occurs from clothing and professional attires. Protection can be numerically expressed as percent Protection Efficiency, i.e., the intercepted fraction of pesticide that *does not* cross the protection and reach the worker's body.

$$\underset{\text{(mg)}}{\text{Skin amount}} = \underset{\text{(mg) * (1-\%shielding efficacy)}}{\text{Deposited Amount * Shielding Efficacy}} \quad (4)$$

Different types of clothing afford different efficiency of personal protection. The highest protection efficiency is afforded by single-use specific professional coveralls; reusable cotton coveralls can afford less protection, and even less does ordinary clothing, often used for working alone. At the lowest end, is working with portions of the body uncovered, as can occur in very hot climates.

Skin absorption of pesticides.

Once the operator's skin has been reached by off-target sprays of pesticide solution, absorption from the skin starts to take place and the active substance enters the body. This fraction builds up and constitutes the body burden. This is the amount of substance that is compared to the occupational limit to calculate exposure-related risk.

$$\underset{\text{(mg)}}{\text{Systemic Amount}} = \underset{\text{(mg) * (\%)}}{\text{Skin Amount * Skin Absorption coefficient}} \quad (5)$$

The skin absorption coefficient is expressed as percent and is usually reported in official databases, but not in the documents delivered to the pesticide user.

The Acceptable Occupational Exposure Limit.

The EU introduced this metric to rank active substances and to calculate exposure-related health risk with the method of dose saturation. This parameter is reported in official databases. Risk assessment (RA) is performed calculating the ratio of the Systemic Amount to the AOEL. The EU regulations assign to the body weight of the subject a default value of 60 kg. Although the actual body weight of the worker can be directly used to improve calculation accuracy, this would not substantially influence the indicative Risk Assessment, due to the effect of default assumptions in the values of the other calculation coefficients.

$$\text{Fractional Risk} \quad = \quad (\text{Systemic amount} / 60) / \text{AOEL} \qquad (6)$$

$$(\%) \qquad\qquad (\text{mg/kgBW}) / (\text{mg/kg}) * 100$$

Appendixes A-C report the tables of the calculation coefficients employed in the current, beta-test version of the described tool.

Agronomical and toxicological characteristics of pesticide Active Ingredients (A.I.) used for Risk Assessment of agricultural use.

This information is reported only as an example. Data has been retrieved from merging information in the following open-source or official databases:

1. PPDB - Pesticides Properties DataBase – AERU (http://sitem.herts.ac.uk/aeru/ppdb/) managed by the Agriculture and Environment Research Unit (AERU) at the University of Hertfordshire. This database does not include Use Rates.
2. EU Pesticides Database - European Commission - Food Safety (https://food.ec.europa.eu/plants/pesticides/eu-pesticides-database_en) as an Excel file (available at: https://ec.europa.eu/food/plant/pesticides/eu-pesticides-database/start/screen/active-substances; last accession for confirmation: 18 December 2024). This database does not include Use Rates.
3. Fitogest (Fitogest: Agrofarmaci e prodotti per un'agricoltura sostenibile [*Agropharmaceuticals and products for sustainable agriculture*]; https://fitogest.imagelinenetwork.com/) collection of product labels for common recommended Use Rates (service available upon time-limited registration)
4. Italian Ministry of Agriculture database and collection of product labels (2022; administrative internal database, not for disclosure) for confirmation of representative or typical values of the Use Rate.

Table S2.1. Agronomical and toxicological characteristics of pesticide Active Ingredients (A.I.) used for Risk Assessment of agricultural use.

	Active Ingredient	Use Rate g/ha [1]	SkP %	AOEL mg/kg_{BW}
**2**	GLYPHOSATE	540	1,00%	0,03
**4**	MANCOZEB	1.500	0,18%	0,035
**3**	PENCONAZOLE	850	3,00%	0,03
**5**	PROPANIL	492	12.40%	0.02
**1**	TEBUCONAZOLE	1.125	13,00%	0,03

[1] representative value calculated as the most frequently reported in the technical document for use on different crops.

Supplementary material 3

Example of application in oliviculture: fighting infestation from the olive mosquito Bactrocera oleae

Figure S2.1 shows the sequential screen output of a pre-application forecast of exposure in the exposure and risk assessment calculation carried out by means of a tablet or notebook. The described simulation is part of a pending exercise based on true-life scenarios.

For use, the farmer enters all the main data regarding the planned activity in Sicurpest and will retrieve a quick, manageable Risk Assessment, as simple three-condition pictogram output: the green "smiling face", for safe application conditions; the yellow "puzzled face" for borderline conditions that may develop into red "scared face", unsafe conditions that make application unadvisable and improvement of working conditions mandatory.

To use the tool, the user will be prompted to upload the information in sequential modules of the app.

In the first one (Figure S3.1a), the user will enter the type of crop, the pest addressed and the total surface to be treated.

The second module (Figure S3.1b), he/she concerns the "Product" that has been suggested by the agricultural consultant or indicated by the local agricultural authorities. In this version of the tool, the user uploads the chemical name of the active ingredient (AI), the type of formulation (liquid, powder, soluble granules and soluble bags) and the concentration of the AI in the product. All data is reported in the product label or mandatory accompanying sheet. Since the exposure coefficients of liquid concentrates and soluble granules resulted similar, and due to the preventive aims of the tool, they have been merged into a single entry.

The third module (Figure S3.1c-d) addresses the selection of personal protective equipment (PPE): mask, headdress, specific or generic gloves, body's protection (normal dresses or generic/specific coverall), feet (shoes or boots). For each type of equipment, it is possible to select "no protection". This section repeats for each of the working phases considered (mixing and loading, application and cleaning and maintenance), since different PPE can be used and separated calculations are performed.

The fourth module (Figure S3.1e) addresses the volume of the application (hectoliters of solution per hectare), the volume of the employed tank (in Liters), the operating pressure and the type of equipment used (open and closed tractor; closed and filtered tractor; manual application with boom; use of knapsack, manual or motorized).

At the end of the data entry phase, the Sicurpest runs the calculation and delivers the Risk Assessment as an emoji which can be green (the forecast is acceptably low risk) yellow s (a borderline situation), or unhappy (the level of risk is not acceptable) (Figure S3.1f).

Trained users access the section of "calculated data", which reports the numeric values that led to the assessment summarized in the emoji. This numeric section allows understanding, in case, in which working phase/s the unacceptable risk has been forecasted, therefore indicating the needed improvements. The user then returns to the data entry module, changes one or more of the critical aspects and runs again the tool to verify the obtained improvement.

Once the results are satisfactory, the document can be saved under the name of the Employer and attached to the risk assessment documents of the farming estate. The responsible farm manager will have the pesticide application started only when the calculated data will show a situation of acceptable risk, and the emoji will be smiling. It is the right of involved employees to be confirmed that their operating conditions are safe, as certified by the INAIL-endorsed Sicurpest calculation tool, before agreeing to start their activity.

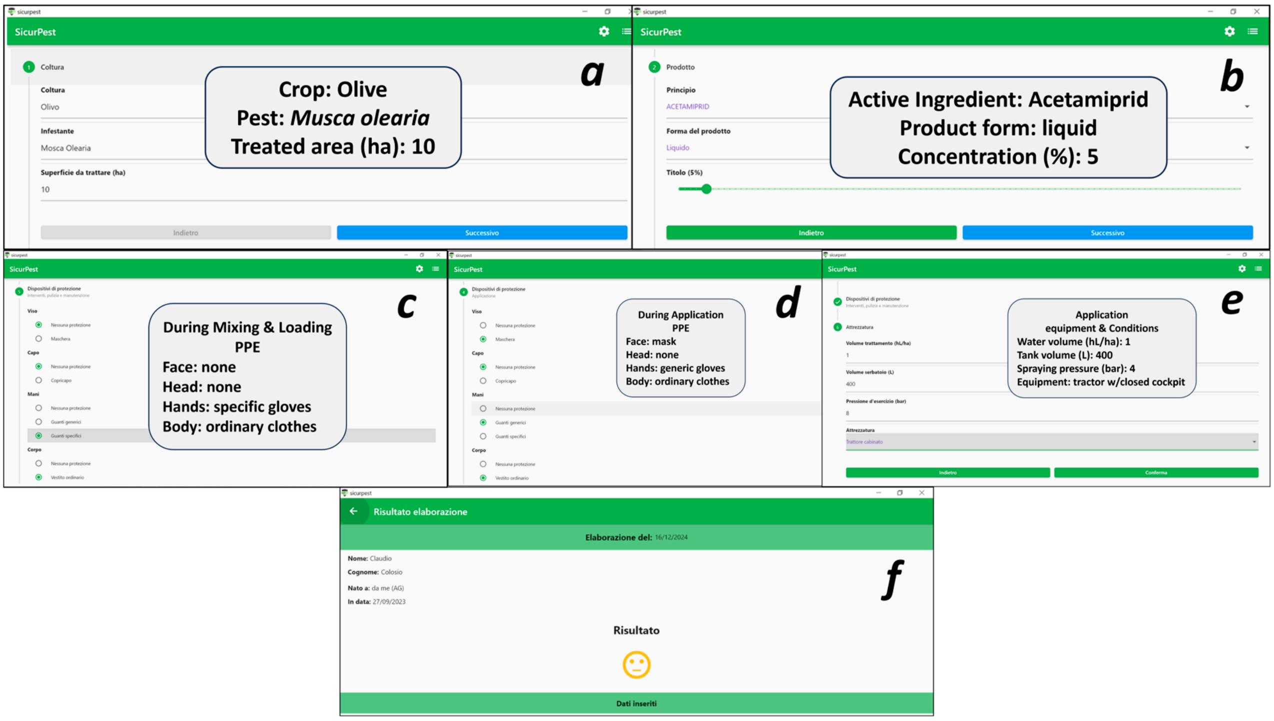

Figure S3.1. The phases of data entry (*a*, *b*, *c*, *d*, *e*) and final risk assessment (*f*) through the tablet version of the Sicurpest tool.
